# The effects of urbanization on bee communities depends on floral resource availability and bee functional traits

**DOI:** 10.1371/journal.pone.0225852

**Published:** 2019-12-02

**Authors:** Caleb J. Wilson, Mary A. Jamieson

**Affiliations:** Department of Biological Sciences, Oakland University, Rochester, Michigan, United States of America; University of Sydney, AUSTRALIA

## Abstract

Wild bees are important pollinators in many ecosystems threatened by anthropogenic disturbance. Urban development can reduce and degrade natural habitat for bees and other pollinators. However, some researchers suggest that cities could also provide refuge for bees, given that agricultural intensification may pose a greater risk. In this study, we surveyed bee communities at 15 farms and gardens across an urban-rural gradient in southeastern Michigan, USA to evaluate the effect of urbanization on bees. We examined how floral resources, bee functional traits, temperature, farm size, and the spatial scale of analysis influence bee response to urbanization. We found that urbanization positively affected bee diversity and evenness but had no effect on total abundance or species richness. Additionally, urbanization altered bee community composition via differential effects on bee species and functional groups. More urbanized sites supported a greater number of exotic, above-ground nesting, and solitary bees, but fewer eusocial bees. Blooming plant species richness positively influenced bee species diversity and richness. Furthermore, the amount of available floral resources was positively associated with exotic and eusocial bee abundances. Across sites, nearly 70% of floral resources were provided by exotic plants, most of which are characterized as weedy but not invasive. Our study demonstrates that urbanization can benefit some bee species and negatively impact others. Notably, *Bombus* and *Lasioglossum (Dialictus)*, were two important pollinator groups negatively affected by urbanization. Our study supports the idea that urban environments can provide valuable habitat for diverse bee communities, but demonstrates that some bees are vulnerable to urbanization. Finally, while our results indicate that increasing the abundance and richness of floral resources could partially compensate for negative effects of urbanization on bees, the effectiveness of such measures may be limited by other factors, such as urban warming.

## Introduction

Managed and wild bee species are threatened by multiple stressors, which have resulted in significant population declines and the extinction of certain species [1–3]. Little is known, however, about the specific population trends of most wild bee species [2]. Because bees are the primary pollinators of most flowering plants, bee declines are predicted to exert economic impacts on agricultural production [3,4] coupled with declines in wild plant diversity and plant community persistence [5,6]. One major cause of bee decline is habitat loss due to anthropogenic disturbance [7,8], and urbanization is a rapidly growing form of such disturbance. Globally, over a million square kilometers of urban land are expected to be added to the earth from 2000–2030 [9].

Some studies have demonstrated negative effects of urbanization on wild bee communities—showing reduced bee abundance, richness, and diversity in urban environments compared to more natural environments [10–14]. However, other studies indicate urbanization may enhance bee abundance and/or species richness [15–17], or that such effects vary across different land uses within a city [18,19]. In certain contexts, cities could support pollinators by providing refuge from agricultural intensification [20]. The influence of urbanization on bee communities, however, is variable and not well-understood [21,22]. Urban bee communities may represent a subset of the regional species pool, dominated by species that thrive in cities [23,24]. Conversely, the increased habitat heterogeneity in cities could support more diverse bee communities than those in more homogenous rural environments [15]. Consideration of how bee abundance, diversity, community composition, and species richness vary across an urban gradient will help explain such contrasting responses.

Urbanization affects pollinators through interrelated direct and indirect effects, including habitat loss and modification, urban warming, and increased exposure to environmental contaminants [25]. In particular, habitat modification reduces floral resource availability and is therefore a key driver of bee response to anthropogenic disturbance [26]. Bee response to such disturbances, however, may differ across functional groups. For instance, removal of floral resources due to urbanization may disproportionately affect bees which nest in the stems of plants by removing both food and nesting resources. Because bees vary in their nesting behavior, sociality, and foraging preferences, their response to urbanization likely depends on their functional traits.

In this study, we investigated how urbanization at the landscape scale and floral resource availability at the local scale influenced wild bee communities at farms and gardens across an urban-rural gradient in southeast Michigan, USA. A central aim of this study was to help inform farmers and gardeners in the metropolitan Detroit area about strategies for supporting pollinators.

In recent decades, urban agriculture has grown in prevalence across the United States and globally [27]. Urban farms and gardens provide numerous social, economic, and public health benefits to their surrounding communities [27–29] and may provide habitat for pollinators [30,31]. Many vegetable and fruit crops grown in urban farms and gardens depend on pollination services provided by bees [32]. Understanding the influence of urbanization on bees and how to mitigate potential negative effects is essential for conserving bee diversity and pollination services in urban environments.

Our primary research objectives were: (1) to examine the effects of urbanization on bee communities, (2) to investigate how functional traits mediate bee response to urbanization, (3) to characterize floral resource availability across sites and evaluate the influence of these resources on bee communities, and (4) to identify which measures of urbanization and floral resources best predict bee response. We hypothesized that urbanization would negatively affect bee communities due to associated habitat degradation and urban warming. Specifically, we expected bee species diversity, richness, and abundance to be lower in more urban areas and that community composition would be simplified. We expected that bee functional traits, including nesting strategy, diet breadth, native or exotic status, and sociality would differentially mediate the effect urbanization had on wild bees. Further, we hypothesized that bloom cover and plant species richness at a site-level would positively influence bee communities, potentially ameliorating the negative effects of urbanization.

## Materials and methods

### Study site characterization

We randomly selected 15 farms and gardens across three counties in southeast Michigan, USA for data collection (S1 Fig, S1 Table). The population estimate for these counties in 2017 was 3.8 million people, making this region the most populous in Michigan [33]. At all sites, farmers and gardeners primarily grew diverse food crops, mostly vegetables and fruits. Wildflowers and/or ornamental flowers were also present at all sites. Our study sites spanned a gradient of urbanization, characterized by the amount of impervious surface in the surrounding landscape and were spaced at least 1500 m apart from each other. Overall, sites were surrounded by 4% to 59% impervious surface area at a 1000 m scale (mean ± SD = 28 ± 19%) and ranged in size from 1,611 to 19,211 m^2^ (mean ± SD = 6,979 ± 4,719 m^2^, S1 Table). Four of our sites were small-scale, for-profit farms (size: mean ± SD = 11,808 ± 5,331 m^2^) and eleven were community gardens (size: mean ± SD = 5,223 ± 3,152 m^2^). Community gardens were characterized as sites where crop cultivation involved community members, who either individually or collectively managed crop production for consumption rather than profit. Our data had a natural break in which approximately half of our study sites had > 40% impervious surface area within a 1000 m radius surrounding each site (mean ± SD = 49 ± 6%) and half the sites had < 40% impervious surface in the surrounding area (mean ± SD = 13 ± 9%, S1 Fig, S1 Table). This relationship was consistent at 1500 m and 2000 m radii and allowed for comparison between less urbanized (< 40% impervious surface) and more urbanized (> 40% impervious) sites at a landscape scale.

To determine the spatial extent of urbanization which best explained variation in bee community and functional group responses at a landscape scale, we measured the proportion of impervious surface at four radii (500 m, 1000 m, 1500 m, and 2000 m) surrounding each study site. We used one-meter resolution aerial imagery from the United States Department of Agriculture’s (USDA) National Agriculture Imagery Program (NAIP) database from 2014 to estimate urbanization. We established site polygons in Google Earth Pro 7.1.8.3036 using aerial imagery from 2017 [34] and created land cover classifications in ArcMap 10.5.1 [35]. Specifically, we created supervised classifications using a maximum likelihood algorithm to delineate impervious surface area from all other land cover classes using false color imagery (band order: short wave infrared, red, blue). For each classified image, we performed post-processing to smooth the resultant classification’s edges and to remove small isolated pixel regions (protocol recommended by 35). We then assessed the accuracy of each classification at 50 randomly stratified accuracy assessment points to ensure correct land cover class assignment. All final kappa accuracy values (a measure of overall classification accuracy) for confusion matrices were ≥ 0.80 [36]. Finally, we estimated urbanization at each spatial scale by dividing impervious surface pixel counts by total pixel counts to calculate the proportion of surrounding impervious surface.

We measured temperature at field sites using two HOBO Pendant temperature loggers (UA-002-08, Onset Computer Corporation, Bourne, MA) per site during the study period from June-August. HOBO loggers recorded temperature measurements every 4 hours. We examined minimum nighttime temperature measurements recorded at each site (before 8:00 and after 20:00) to avoid issues with measurement error associated with the influence of solar radiation on data loggers [37–39]

### Floral resource surveys

We conducted floral and bee community surveys across our 15 study sites in June, July and August of 2017. To evaluate the influence of floral resource availability on bee response variables, we examined two metrics of floral resources at the site level: bloom cover and blooming plant species richness. To obtain these floral metrics, we inventoried plants along four 25 m transects, which were haphazardly placed to capture a representative sample of the abundance and diversity of available floral resources. We inventoried floral resources provided by trees or shrubs only when they intersected transects, which was infrequent for shrubs and did not occur for trees. Thus, floral resources described in our study are largely herbaceous species. All surveys were conducted within 100 m of the center of each site. Within one meter from the centerline of each survey transect, we identified plants in bloom to the lowest taxonomic level possible. We estimated bloom cover for each species as a percentage of the entire transect area (50 m^2^), using a 1 m^2^ and 0.05 m^2^ PVC quadrat. Then, we converted bloom cover percentages to square meter estimates and summed the total bloom cover recorded along transects to generate a site-level estimate of bloom cover. We estimated blooming plant species richness at the site-level by summing the number of species in bloom across transects. Across sites, bloom cover and blooming plant species richness were not influenced by urbanization (e.g., at a 1000m radius, bloom cover: R^2^ = 0.10, t_13_ = -1.262, P = 0.229 and plant species richness: R^2^ = 0.02, t_13_ = -0.474, P = 0.643).

Plants were categorized based on species origin (native, exotic, or uncertain) using the United States Department of Agriculture’s Plants database [40]. We further classified plants into one of three groups: noxious weeds, wildflowers, and cultivated plants (ornamentals and crops). We classified noxious weeds according to definitions and classifications provided by the USDA Plants Database and the USDA Natural Resources Conservation Service (NRCS) [40,41]. Cultivated plants include cultivated varieties of native or exotic plants and all crops. Wildflowers were non-cultivated native or exotic plants that were not classified as noxious weeds by referenced USDA sources.

### Bee community surveys

To sample bees across study sites, we used a combination of pan traps and hand-netting to reduce bias from utilizing either capture method alone [42]. We sampled bees three times across the summer of 2017. The sampling periods ran from June 7—June 14, from July 3—July 8, and from July 31—Aug 8. We placed four pan trap triplets at each study site during the first survey period, and then six pan traps for the final two survey periods due to initially low numbers of collected bees. During each survey period triplets were collected after 48 hours. Pan trap triplets consisted of three 150ml plastic cylinders colored fluorescent white, yellow, and blue held in place with a PVC base and a bamboo stake. Each trap was filled 3/4ths full of soapy water (Dawn Ultra Liquid Dish Soap, Procter & Gamble, Cincinnati, OH.). We placed pan traps near patches of blooming flowers at each site so that each bowl was at least 5 m apart from all others. Traps were placed to sample bees near all major patches of floral resources while also covering the entire extent of each site.

During each survey period, we sampled bees via hand-netting along floral survey transects. We walked along each transect for seven and a half minutes, thus sampling for 30 minutes at each field site during each sampling period, per recommended guidelines [43]. During each survey period, sampling was split up between two observers so that each person sampled along two transects. We did not count handling time spent transferring bees into collection vials. During the final survey period, we counted rather than collected honey bees (*Apis mellifera* Linn.). We sampled bees only on days that met the following criteria: (1) ambient temperatures were greater than 15.5 degrees C, (2) wind speeds less than 3.9 mps for 30 seconds, and (3) enough continuous sunlight to cast a shadow [guidelines modified from 44]. Sites were sampled in different order by different surveyors across survey periods. All collected bees were identified to species or morphospecies. Specimens of taxonomically challenging genera were identified or verified by Rob Jean (Environmental Solutions & Innovations), Jason Gibbs (University of Manitoba), and Karen Wright (Texas A&M University). See S2 Table for a complete list of collected bee species.

Because the goal of our study was to evaluate the influence of urbanization and floral resources on wild bee species, we excluded the European honey bee (*Apis mellifera*) from analyses as has been done in other studies [13,45,46]. Since *A*. *mellifera* is a managed species, the distribution and abundance of individuals is influenced by placement and management of hives, which could not be adequately assessed across our study sites. While research indicates that honey bees can have competitive effects of on wild bees [47], we found no significant relationship between honey bee and wild bee abundance (R^2^ = 0.058, t_13_ = 1.065, P = 0.306). Additionally, we found no relationship between urbanization and honey bee abundance (at a 1000 m radius: R^2^ = 0.058, t_13_ = -1.742, P = 0.389). Descriptive data on honey bee abundance in comparison to wild bee abundance across sites are presented for honey bees, but analyses focus on wild bee data.

### Bee community traits and functional groups

To evaluate the influence of urbanization and floral resources on bee communities across sites, we examined four bee community response variables: total abundance, species richness, diversity, and evenness. We used individual-based rarefaction to estimate species richness based on 100 randomizations using EstimateS 9.1.0 [48]. This statistical interpolation method accounts for uneven sample size and standardizes estimates of species richness across samples [49]. From rarefied species richness data, we calculated the exponential form of Shannon entropy. This species diversity metric produces an estimate that transforms Shannon entropy into an “effective number of species” and provides a more stable and interpretable measure of diversity [50]. Finally, we calculated Pielou’s evenness index (J) to compare the relative evenness of species abundances across sites [51].

To examine how functional groups mediate bee response to urbanization, we sorted species based on the following traits: nesting strategy (ground, cavity, stem, soft wood, hard wood, managed hives), diet breadth (generalist—collecting pollen from many plant families, specialist—collecting or preferring pollen from one plant family), native or exotic status, and sociality (solitary, communal, subsocial, eusocial, cleptoparasite). We assigned specimens to functional group categories using information sourced primarily from Gibbs et al. [52] and references therein. Species that use multiple nesting substrates or social strategies were assigned to the category they most frequently use based on regional records. For a complete list of species collected and functional group assignments, see S2 Table. For analyses, we combined bees that nest in stems, cavities, and wood into an above-ground nesting group. Similarly, solitary, communal, subsocial, and cleptoparasitic bees were combined into a single category—solitary bees. Primitively eusocial Halictidae and eusocial bumble bees were grouped as eusocial bees. *Lasioglossum (Dialictus)* species were treated as eusocial when records were lacking because this group is ancestrally eusocial and most studied species have been shown to make eusocial nests [53–55]. In instances where no functional trait information was available, specimens were assigned to functional groups based on information known for closely related taxa (i.e. known traits for species within a morphospecies group, e.g. *Hylaeus modestus* group, or assumed genus-level traits). For example, many *Andrena* species do not have published records of nesting behavior, but most studied species are known to nest in the ground. Therefore, ground-nesting behavior was inferred for all *Andrena* when no published records existed. In total, we used this approach to infer functional traits for 29 species and 8 morphospecies. When information was lacking and could not be adequately assessed, we excluded species from analyses. Functional group assignments, including instances of inferred traits and exclusions are listed in S2 Table.

### Statistical analyses

Statistical analyses were performed in R v. 3.5.2 [56]. We used general linear models to examine the influence of urbanization and floral resources on bee community response variables and bee functional group abundances at the site level with temperature and farm size included as covariates. We ln(x+1) transformed bee response variables to meet assumptions of statistical tests. We transformed data for model fitting instead of modelling relationships with a count distribution to prevent inflating type-I error given our small sample size [57].

We used a forward selection approach to choose predictor variables and models with the lowest AICc. First, we selected the urbanization radius which best explained bee response to urbanization (impervious surface from 500 m, 1000 m, 1500 m, 2000 m) by fitting a model that included each variable as a single predictor. Next, we compared models which contained the best urbanization predictor from the first step and all combinations of our floral resource predictors (total bloom cover & plant spp. richness together, and separately). We then took the resultant best model and assessed whether the addition of farm size and temperature improved the fit of each model. This process was repeated for all bee response variables. We evaluated the significance of predictors using type-II F-tests.

To examine the composition of bee species and functional groups across sites and urbanization categories, we used non-metric multidimensional scaling (NMDS) via the R package “vegan” [58]. Bee species and functional group data were square root transformed and then Wisconsin double standardized before calculating Bray-Curtis dissimilarity measures. This was done to reduce stress in the final ordination [59]. We constructed polygons on ordination plots to delineate sites belonging to each urbanization category. We conducted an Analysis of Similarities (ANOSIM) permutation test, set to 999 permutations, to determine if the composition of species and functional groups differed significantly for the two urbanization categories. Finally, we performed a Mantel test using Pearson’s correlation coefficient to determine if bee species and functional group composition were spatially autocorrelated. We used latitude and longitude measurements to calculate geographic dissimilarity of sites based on Euclidean distance which were compared to species and functional group Bray-Curtis dissimilarity matrices.

## Results

**Fig 1:**
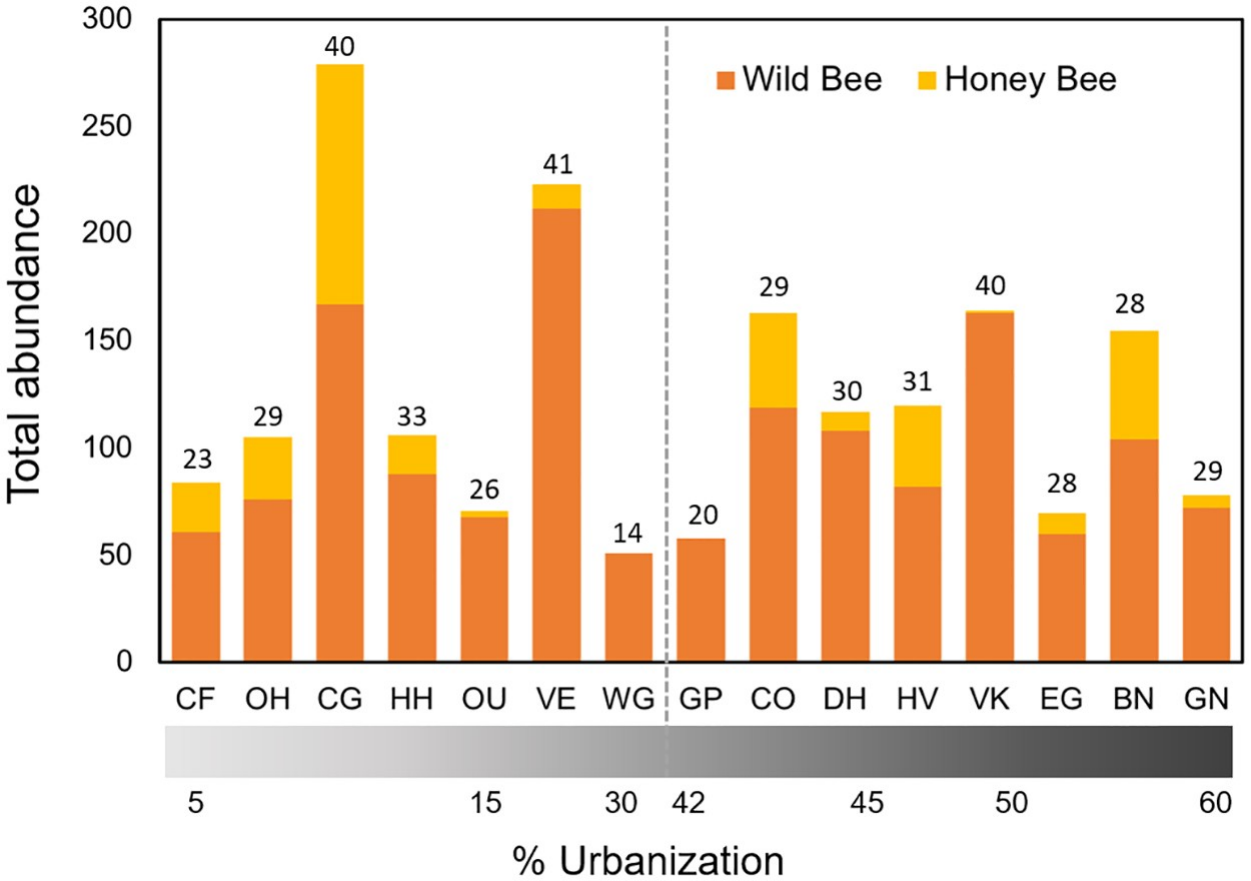
Bee abundance (bars) and species richness (numbers above bars) across study sites. Sites are ordered by increasing urbanization with values for % urbanization (measured as impervious surface) shown along the bottom of the x-axis. Urbanization values are provided in S1 Table. For some analyses, sites were divided into two categories: less urbanized (mean ± SD = 13 ± 9) and more urbanized (mean ± SD = 49 ± 6). The dashed gray line illustrates how sites were divided with associated levels of urbanization.

In total, we collected 1,844 bee specimens representing 106 species and 12 morphospecies from 26 genera within five families (S1 and S2 Tables, Fig 1, S2 and S3 Figs). Of these specimens, 355 were honey bees (*Apis mellifera* Linn.). Next to *A*. *mellifera*, the most abundant bee species was *Bombus impatiens* Cresson with 140 specimens (9%) followed by *Lasioglossum imitatum* (Smith), *Halictus ligatus* Say, *Ceratina calcarata* Robertson, and *Hylaeus hyalinatus* Smith (respectively 7%, 4%, 3%, and 3% of the bee community; S4 Fig). The number of wild bee species observed across sites ranged from 14 to 41 (mean ± SD = 29.4 ± 7.4; S1 Table).

The majority of collected bee species were ground-nesters (63.9%). Above-ground nesters (36% of all bees) consisted of cavity (22%), stem (11%), and wood-nesting bees (3%). Most wild bee species were either eusocial (46%) or solitary (43%). Subsocial and communal bees comprised 7% and 3% of all species, respectively. For the remaining functional groups, we found more generalist species (89%) compared with specialists and native bee species (87%) compared with exotic. Since bees were characterized by multiple traits, certain functional groups showed considerable overlap. In particular, all exotic and cavity-nesting bees were solitary, 77% of exotic bees were cavity-nesting, and 98% percent of eusocial bees were ground-nesting.

Most flowering species available at our sites were exotic species (68%), representing 66% of total bloom cover (S3A Table). Wildflowers were the most abundant plant category representing 56% of plant species and 54% of total bloom cover. Forty two percent of plant species were ornamentals or crops, constituting 35% of overall bloom cover. Noxious weeds comprised approximately 2% of species and 11% of the total bloom cover across sites and dates (S3B Table). In order of abundance, the five most commonly recorded plant species were *Rudbeckia hirta* L., *Daucus carota* L., *Erigeron spp*. L., *Achillea millefolium* L., and *Trifolium repens* L.. Two of these species (*D*. *carota* and *T*. *repens*) are naturalized wildflowers from Eurasia that are considered to be weedy species, but not noxious or invasive.

### Effects of urbanization and floral resources on bee community traits

**Fig 2:**
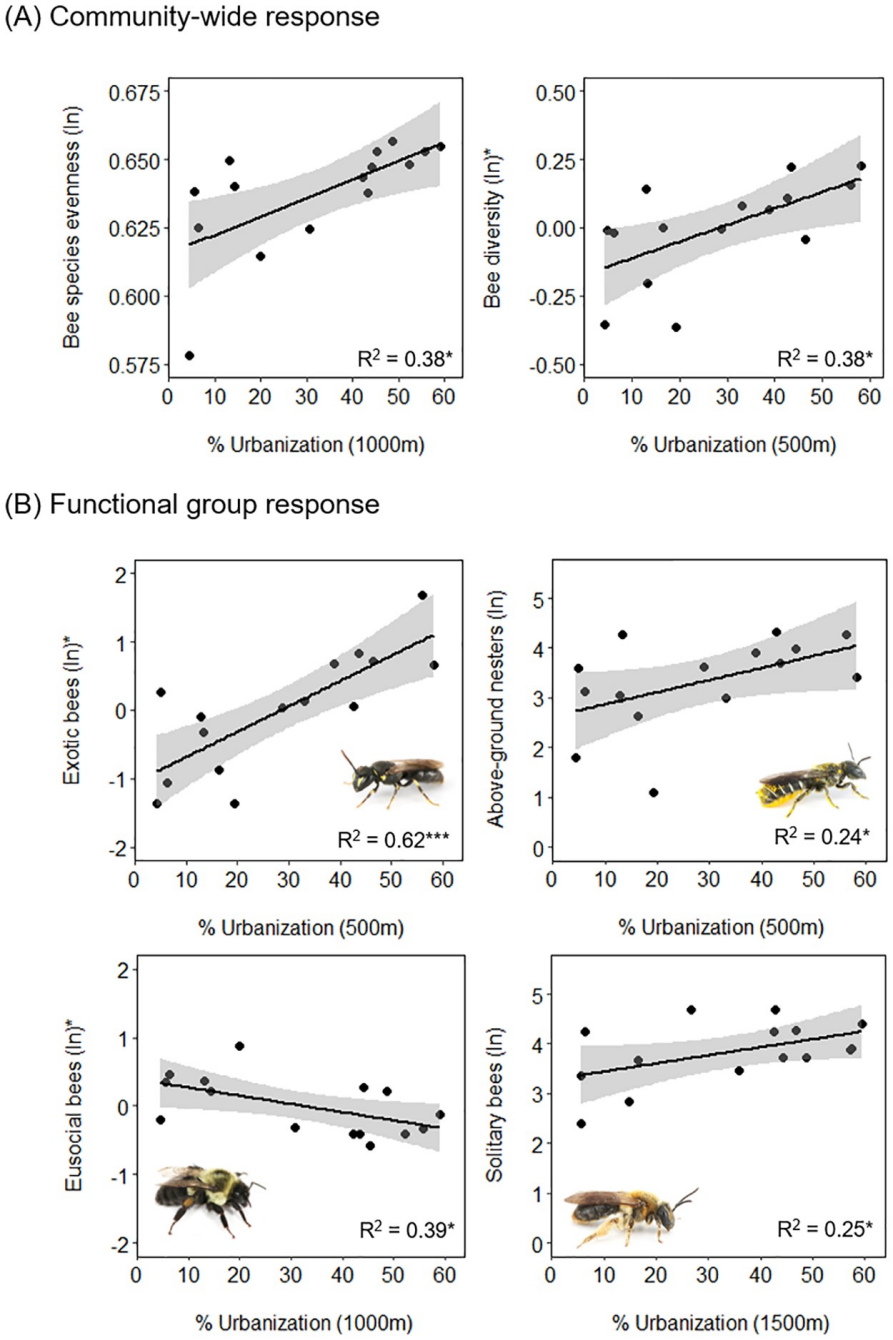
Relationships between wild bee response variables and urbanization predictors. Ln(x+1) transformed data are plotted on all y-axes except those with asterisks, which indicate partial residuals are plotted instead. Partial residuals show the remaining variation explained by the plotted predictor variable when the remaining variation explained by another predictor is accounted for. Partial residuals are plotted in instances where final models had two significant predictors (Table 1). Asterisks next to R^2^ values indicate the significance of the plotted relationship: * P < 0.05, *** P< 0.001.

**Fig 3:**
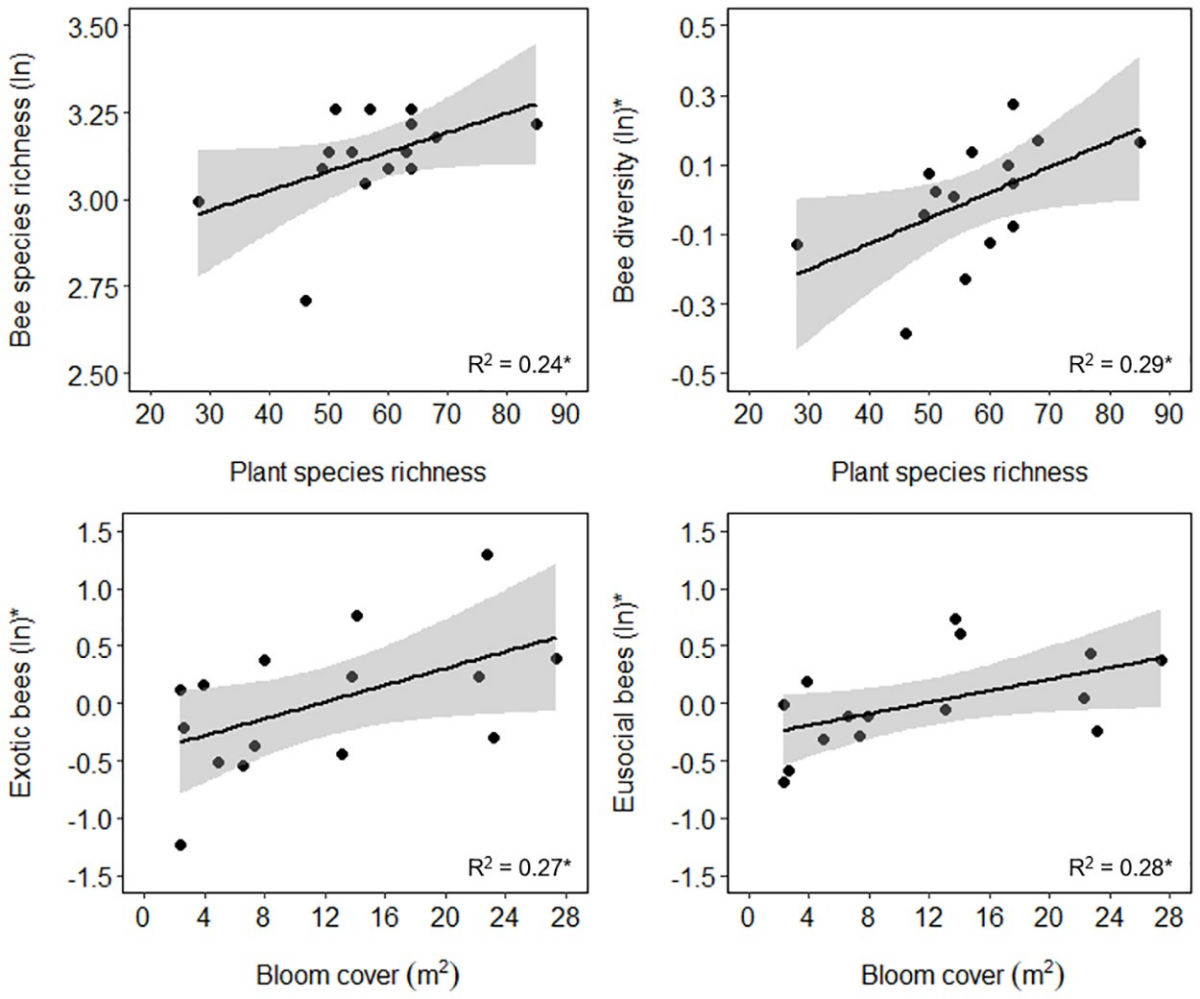
Influence of floral resources on wild bee response variables. Ln(x+1) transformed data are plotted on all y-axes except those with asterisks, which indicate partial residuals are plotted instead. Partial residuals were plotted for groups which had two significant predictor variables in final models (Table 1). Partial residuals show the remaining variation explained by the plotted predictor variable. Asterisks next to R^2^ values indicate the significance of the plotted relationship: * P < 0.05.

Urbanization positively influenced bee species diversity and evenness but had no effect on bee abundance or species richness (Fig 2A; Table 1A). Urbanization explained the most variation in bee diversity at a 500 m radius (Fig 2A) and in species evenness at a 1000 m radius (Fig 2A). For these analyses, model fits were comparable across spatial scales (ΔAICc < 2; S4A Table). Greater plant species richness positively influenced bee diversity and species richness (Fig 3). However, no floral resource metrics significantly affected bee species evenness or abundance (Table 1A).

**Table 1:** (A) Bee community and (B) functional group response to urbanization, temperature, floral resources, and farm size.

Response Variable	Parameters	Multiple R^2^	Estimate	SE	F	DF	P
*A*. *Community level*							
Total bee abundance	Urbanization (1500m)	0.171	0.124	0.587	0.044	(1,12)	0.837
	Bloom cover		0.021	0.013	2.432	(1,12)	0.145
Bee species richness	Urbanization (500 m)	0.363	0.254	0.174	2.121	(1,12)	0.171
	**Plant species richness**		**0.006**	**0.003**	**5.468**	**(1,12)**	**0.038** *
Bee evenness	**Urbanization (1000m)**	**0.472**	**0.070**	**0.022**	**10.395**	**(1,12)**	**0.007** *
	Plant species richness		0.001	0.001	1.425	(1,12)	0.339
Bee diversity	**Urbanization (500m)**	**0.486**	**0.617**	**0.223**	**7.658**	**(1,12)**	**0.017** *
	**Plant species richness**		**0.007**	**0.003**	**5.121**	**(1,12)**	**0.043** *
*B*. *Functional groups*							
Native bees	Urbanization (1000m)	0.186	0.244	0.125	0.169	(1,12)	0.688
	Total bloom cover		0.019	0.012	1.900	(1,12)	0.193
Exotic Bees	**Urbanization (500m)**	**0.666**	**3.908**	**0.820**	**22.684**	**(1,12)**	**< 0.001** ***
	**Total bloom cover**		**0.039**	**0.018**	**4.771**	**(1,12)**	**0.050** *
Ground-nesting bees	Urbanization (1000m)	0.420	1.024	0.550	3.472	(1,12)	0.087
	Total bloom cover		0.019	0.013	2.378	(1,12)	0.149
Above-ground nesting bees	**Urbanization (500m)**	**0.345**	**2.643**	**1.161**	**5.177**	**(1,12)**	**0.042** *
	Plant species richness		0.023	0.017	1.844	(1,12	0.199
Solitary bees	**Urbanization (1500m)**	**0.361**	**2.211**	**0.851**	**6.743**	**(1,12)**	**0.023** *
	Total bloom cover		0.009	0.020	0.218	(1,12)	0.649
Eusocial bees	**Urbanization (1000m)**	**0.596**	**1.348**	**0.529**	**6.505**	**(1,12)**	**0.025** *
	**Total bloom cover**		**0.028**	**0.012**	**5.380**	**(1,12)**	**0.039** *
Generalist bees	Urbanization (1500m)	0.206	0.050	0.624	0.007	(1,12)	0.937
	Total bloom cover		0.025	0.014	2.956	(1,12)	0.111
Specialist bees	Urbanization (500m)	0.171	1.290	0.860	2.248	(1,12)	0.160
	Plant species richness		0.004	0.013	0.082	(1,12)	0.779

Response variables were ln(x + 1) transformed for analyses. Multiple R^2^ values are reported for whole models. Model parameter significance is indicated as:

* P < 0.05,

*** P < 0.001.

**Fig 4:**
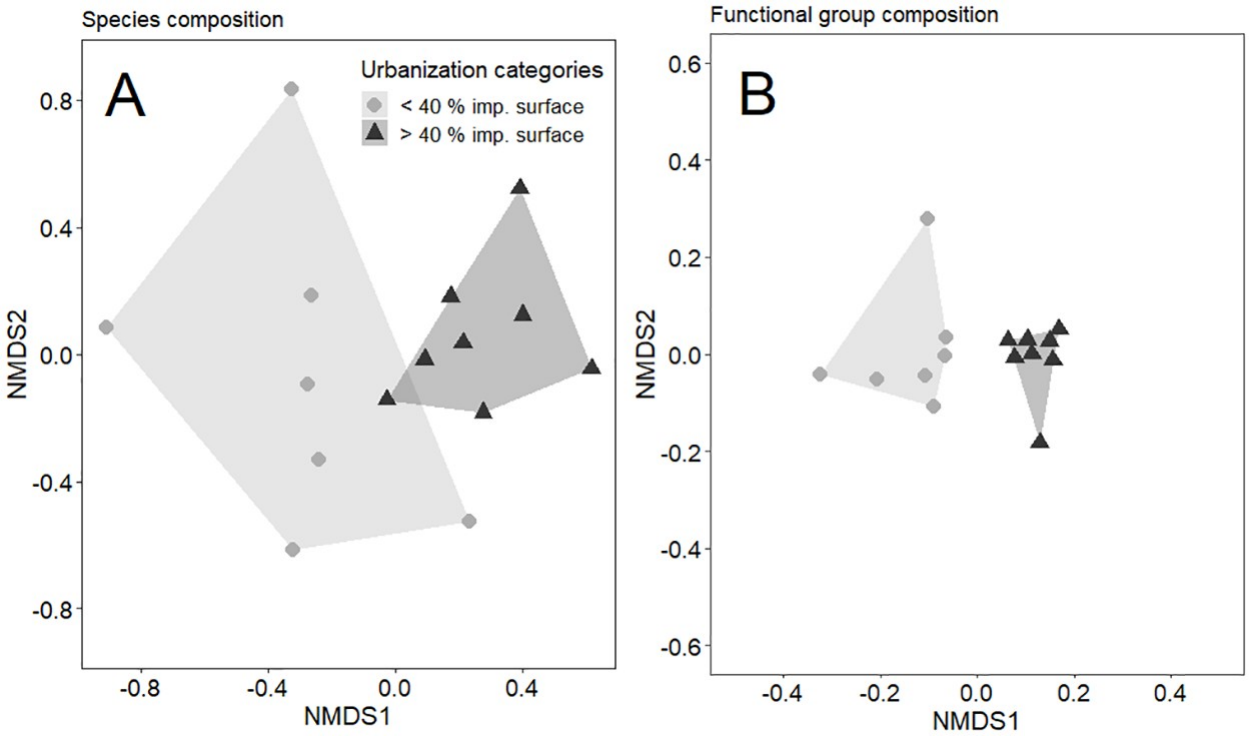
Non-metric multi-dimensional scaling (NMDS) ordinations of (A) Bee species and (B) functional group composition across study sites (N = 15). Less urbanized sites have < 40% while more urbanized sites have > 40% surrounding impervious surface area at a 1000 m radius. Polygons show clustering of sites within each urbanization category in reduced ordination space. The composition of less urbanized and more urbanized sites was significantly different for both ordinations. Stress was 0.166 for (A) and 0.033 for (B).

Bee species and functional group composition differed significantly between more urbanized and less urbanized sites (ANOSIM: R = 0.217, 0.352; P = 0.038, 0.001; Fig 4). More urbanized sites clustered closely together and thus were more similar in bee species and functional group composition compared with less urbanized sites (Fig 4). Species composition, however, was spatially autocorrelated (Mantel test: r = 0.444, P = 0.001), while functional group composition was not (Mantel test: r = 0.084, P = 0.233).

### Effects of urbanization and floral resources on functional groups

Urbanization positively influenced the abundances of exotic, above-ground nesting, and solitary bees, whereas eusocial bees were negatively affected (Fig 2B; Table 1B). Urbanization at a 500 m radius provided a significantly better model fit for exotic bees (S4B Table). For ground-nesting and eusocial bees, larger spatial scales of analysis (1000-2000m) provided better model fits for examining the effect of urbanization on these functional groups (S4B Table).

Because eusocial bees were the only functional group negatively affected by urbanization, we conducted separate post-hoc analyses on bees in the two most abundant genera—*Bombus* and *Lasioglossum (Dialictus)*—which represent 77% of eusocial bees in our study. Since *Bombus* and *Lasioglossum (Dialictus)* differ greatly in size, and thus foraging ability, we expected that these genera could respond differently to urbanization. Post-hoc analyses, however, showed that both genera were negatively influenced by urbanization (S5 Fig), and that all urbanization radii provided similar model fits (S4C Table). In general, statistical models using different urbanization radii were comparable for most functional groups (ΔAICc < 2), unless otherwise noted above (S4 Table).

Overall, blooming plant cover was a better floral resource predictor for bee functional group response in comparison to plant species richness (Table 1B, S4B Table). However, this floral resource metric only positively influenced eusocial bee abundance and exotic bee abundance (Table 1B; Fig 3).

Neither site-level temperature nor farm size improved the fit of any community level or functional group models (S4 Table). Urbanization and minimum temperature, however, were positively correlated, and thus community level and functional group responses to urbanization cannot be disentangled from bee response to warmer temperatures associated with greater urbanization (S5 Table).

## Discussion

Because some bee species and functional groups respond differently to anthropogenic drivers, detecting effects on the overall community is challenging [21]. A growing body of research, however, has found that bee functional traits can be used to help understand how bees respond to the effects of anthropogenic changes, such as urbanization [60–64]. Across our study region, more urbanized sites supported a greater number of above-ground nesting, exotic, and solitary bees. Ultimately, the positive response of these particular functional groups explain the overall positive effects of urbanization on bee diversity and evenness observed in our study. Other studies have also shown that cavity-nesting and exotic bees are often more abundant in cities [reviewed in 22,25], which suggests that urbanization could homogenize bee communities by preferentially supporting these groups. Indeed, a recent study showed that urban landscapes were associated with phylogenetic homogenization of bee communities across ecoregions in the northeastern United States [46]. Thus, although some urban areas, such as our study region, can support diverse bee communities, these habitats may be dominated by certain functional or phylogenetic groups.

While some functional groups responded positively to urbanization in our study, many were unaffected and eusocial bees were negatively affected. The negative effect of urbanization on eusocial bees is especially concerning given their ecological and economic importance. Bumble bees, in particular, are key pollinators for many plant species, including a number of fruit and vegetable food crops [65]. Given that many bumble bee species are experiencing widespread decline [1,66–68], conservation measures targeting these species are critical for buffering against further losses.

In our study, bloom cover was an important predictor of eusocial bee abundance, indicating that speciose and abundant urban wildflower plantings are needed to support these species. Bee reproductive success is influenced by the availability and proximity of floral resources [69,70], and bees prefer to nest close to areas of ample bloom cover [71]. Thus, if floral resources are locally dense within cities, bees would not have to forage far to collect necessary resources for colony survival. Greater floral resource abundance and diversity has been shown to positively affect the fitness and reproductive output of eusocial bee colonies [72]. Furthermore, local scale floral resource additions to an urban area have been linked with increased bee density and abundance [73,74].

Our study supports previous research showing that increased floral resource availability can enhance bee diversity and abundance in urban environments [23,63,75–78]. We found that bee diversity and species richness were positively influenced by blooming plant species richness. These results suggest that increasing flowering plant diversity is an important conservation measure. Many of the floral resources found across our sites were exotic species, mostly naturalized weedy wildflowers. Such species can provide important floral resources for bees in areas and at times when native species are lacking [79–81]. However, while wild bees will forage on exotic plants, they often prefer native species [81–83]. Given the value of native plants in supporting arthropod biodiversity [84–86] and the dominance of exotic flora in our study region and other urban ecosystems [77,87–89], we recommend conservation efforts encourage planting more native species to support urban bees.

Interestingly, some researchers have found positive effects of urbanization on bumble bees [90–92], while others have found declines with greater urbanization [11,93]. Similarly, Harrison et al. [46] found that *Lasioglossum* were more abundant at urban study sites compared with agricultural and forested sites, whereas we found a negative effect of urbanization on *Lasioglossum*. Collectively, these context-dependent results indicate a need for research across broad geographic regions as well as a better understanding of how contextual differences contribute to variable results. In some cases, variable results may arise due to differences in study methodologies or habitat surveyed. For example, Harrison et al. [46] compared *Lasioglossum* in three discrete land-cover categories, while our study examined *Lasioglossum* across similar habitats that varied in the amount of urbanization in the surrounding landscape. In Harrison et al., sites were characterized as at least 80% urban, agriculture, or forest, whereas the degree of urbanization ranged from 5–60% in our study. Furthermore, in our study, rural sites were open habitats similar to urban and peri urban sites and were not predominately agricultural. Thus, rural sites in our study area may have been better at supporting *Lasioglossum* in comparison to our urban sites, yielding results that differ from the findings of Harrison et al. Variation in sampling technique, survey period, and spatial scale of analysis are other methodological differences that could underlie inconsistent results.

In our study, the spatial scale which explained the most variation in bee response varied across groups and between community metrics. However, all spatial scales explained similar amounts of variation (ΔAICc < 2), except in the case of exotic bees (best radius 500 m), ground nesting bees (best radii > 500 m), and eusocial bees (best radii > 500 m). Similarly, bee diversity was the only community metric with large difference in model fit from one radius compared to all others (500 m). Some researchers have examined bee response to urbanization at smaller spatial scales [e.g. 13], while other use several scales [e.g. 93], and responses across radii are often similar. Our results suggest that for most functional groups, impervious surface extent measured at large and small spatial scales will effectively approximate bee response to urbanization.

We found no effect of increased urban temperatures on bee response variables after accounting for the effects of impervious surface area, Heat island effects vary by latitude and are generally expected to produce positive effects on species at higher latitudes [94]. Given the location of our study area, the warmer temperatures of our urban study sites may still be within the range of optimal growth and development for many bee species. The effects of greater impervious surface on habitat loss and urban warming, however, cannot be disentangled in our study. Nonetheless, some bee species are more sensitive to hotter temperatures than others, and thus may be negatively affected by urban warming [39,95]. Thus, while efforts to increase floral resource abundance and diversity will certainly benefit many urban bees, these conservation measures will likely not ameliorate other negative effects of urbanization—such as the effects of increased impervious surface on warming trends and the availability of nesting sites for ground-nesting bees.

For most of the world's wild bee species, we know little about how species' ranges and populations are changing with urbanization, and what we do know is limited in scope across geographic areas [96,97]. Our study adds to the knowledge of bees in southeastern Michigan, contributing information to an area that has not been historically well-studied. Overall, this project added new state occurrence records for two exotic bee species as well as 74 new county-level species records [96]. Further research, especially multi-year studies across broad geographic regions, are needed to help monitor and mitigate the effects of urbanization and other human-caused environmental changes on bee communities.

As cities continue to grow worldwide, effective management strategies must be developed to support bees and the important pollination services they provide. City and land managers can work alongside conservationists, farmers, and gardeners to support urban biodiversity. Critical components of pollinator conservation in cities include the development and maintenance of habitats that provide diverse and abundant floral resources in addition to nesting substrates while limiting detrimental effects of urban warming and environmental pollutants. Such considerations are essential in urban development and urban agriculture.

## Supporting information

S1 Fig Map of study sites in southeastern Michigan, USA. (DOCX)

S2 Fig Rank abundance of bee genera ordered by total number of specimens observed across sites. (DOCX)

S3 Fig Bee abundance and species richness across sites and sampling periods. (DOCX)

S4 Fig The most abundant wild bee species collected across sites. (DOCX)

S5 Fig Effect of urbanization on *Bombus* and *Lasioglossum (Dialictus)* abundance. (DOCX)

S1 Table Site-level bee community data. (DOCX)

S2 Table Summary of collected bee specimens with functional trait data. (DOCX)

S3 Table The species number and bloom cover provided plants of different geographic origin and plant category. (DOCX)

S4 Table Summary of AICc values for model selection. (DOCX)

S5 Table Correlation tests between minimum site level temperature and impervious surface area. (DOCX)
