# Supplementary material for: The effects of urbanization on bee communities depends on floral resource availability and bee functional traits
Source: PLoS One. 2019 Dec 2;14(12):e0225852. doi: 10.1371/journal.pone.0225852 (PMC6886752; doi:10.1371/journal.pone.0225852)
Supplement: S1 Fig — (DOCX) [file pone.0225852.s001.docx]

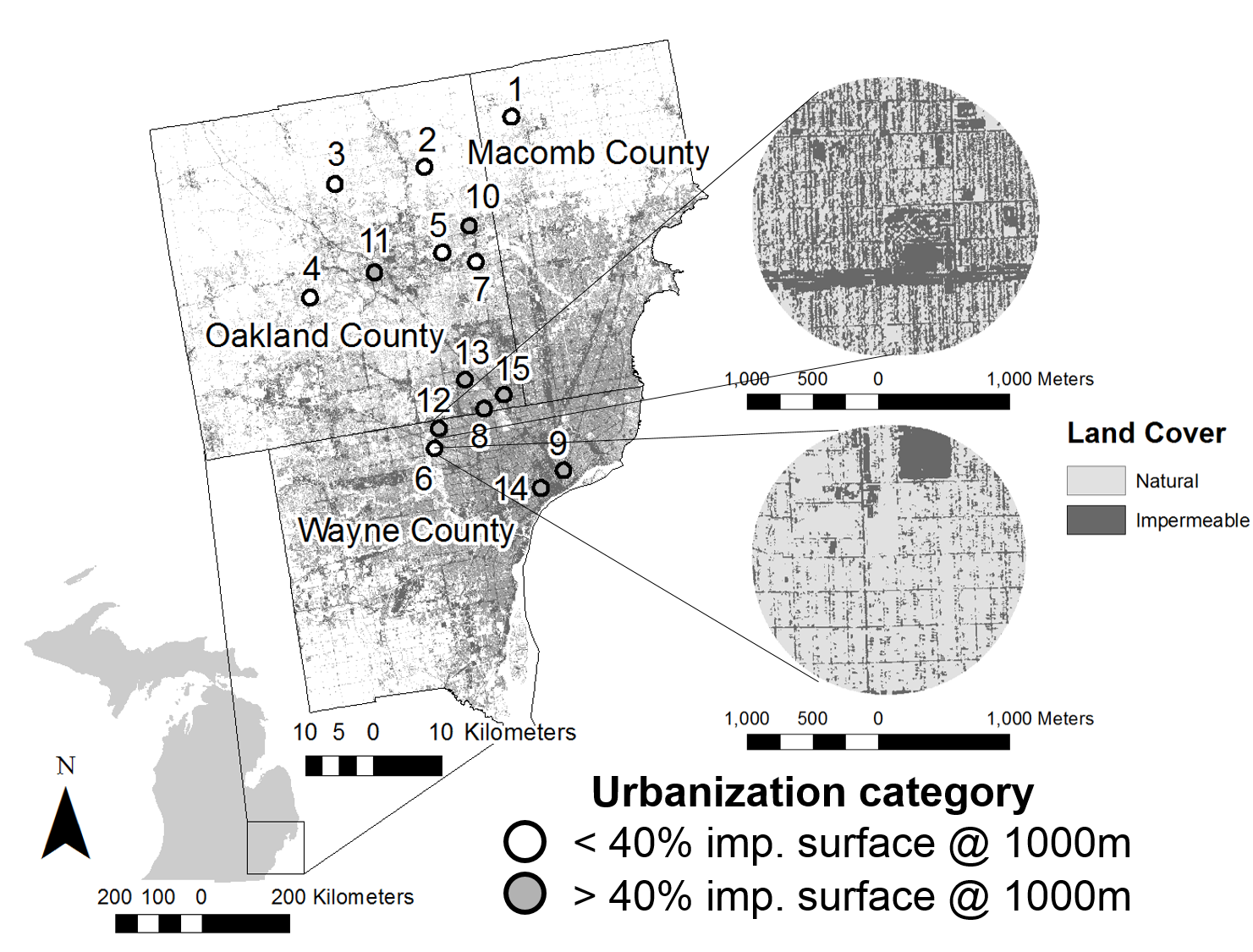


S1 Figure. Map of study sites in southeastern Michigan, USA. The state of Michigan is shown in the bottom left corner of the figure. Urbanization categories at the 1000 m radius are shown. Urbanization was estimated as the percent impervious (imp.) surface in the surrounding landscape at four concentric radii (see Table S1). Sites are enumerated in order of increasing urbanization at a 1000 m radius and correspond to site numbers in Table S1
