# Supplementary material for: The effects of urbanization on bee communities depends on floral resource availability and bee functional traits
Source: PLoS One. 2019 Dec 2;14(12):e0225852. doi: 10.1371/journal.pone.0225852 (PMC6886752; doi:10.1371/journal.pone.0225852)
Supplement: S2 Fig — (DOCX) [file pone.0225852.s002.docx]

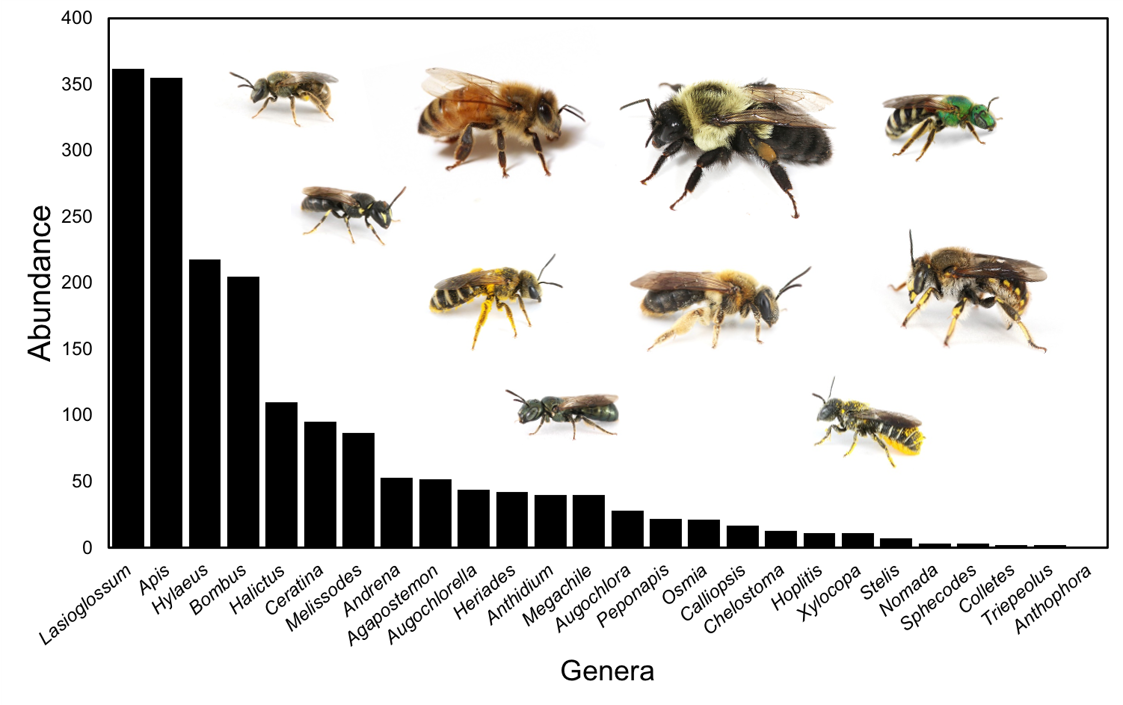


S2 Figure. Rank abundance of bee genera ordered by total number of specimens observed across sites. Images of bees show species of the most abundant genera. Photos provided by Joseph Wilson
