# Supplementary figures and images for: The effects of urbanization on bee communities depends on floral resource availability and bee functional traits
Source: PLoS One. 2019 Dec 2;14(12):e0225852. doi: 10.1371/journal.pone.0225852 (PMC6886752; doi:10.1371/journal.pone.0225852)

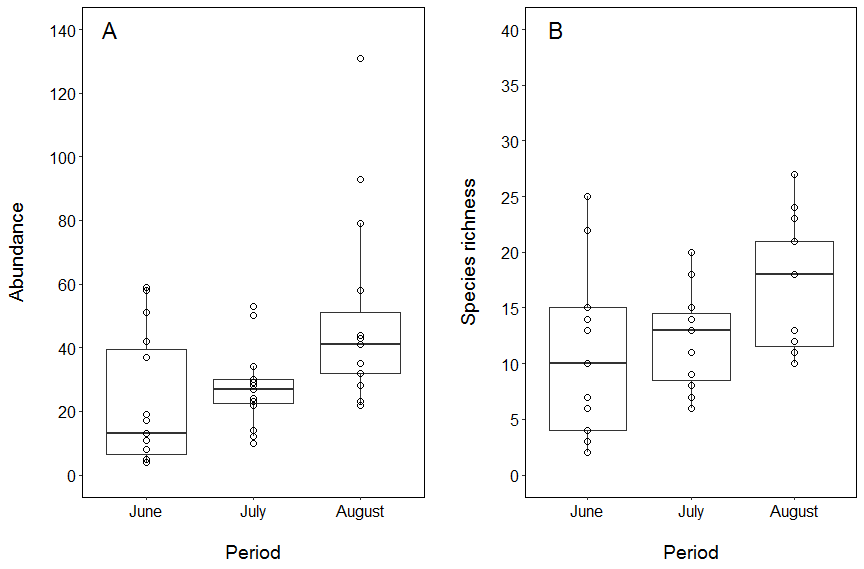


S3 Figure. (A) Bee abundance and (B) species richness across sites (N = 15) and sampling periods

Supplement: S3 Fig — (DOCX) [file pone.0225852.s003.docx]
