# Supplementary material for: The effects of urbanization on bee communities depends on floral resource availability and bee functional traits
Source: PLoS One. 2019 Dec 2;14(12):e0225852. doi: 10.1371/journal.pone.0225852 (PMC6886752; doi:10.1371/journal.pone.0225852)
Supplement: S4 Fig — (DOCX) [file pone.0225852.s004.docx]

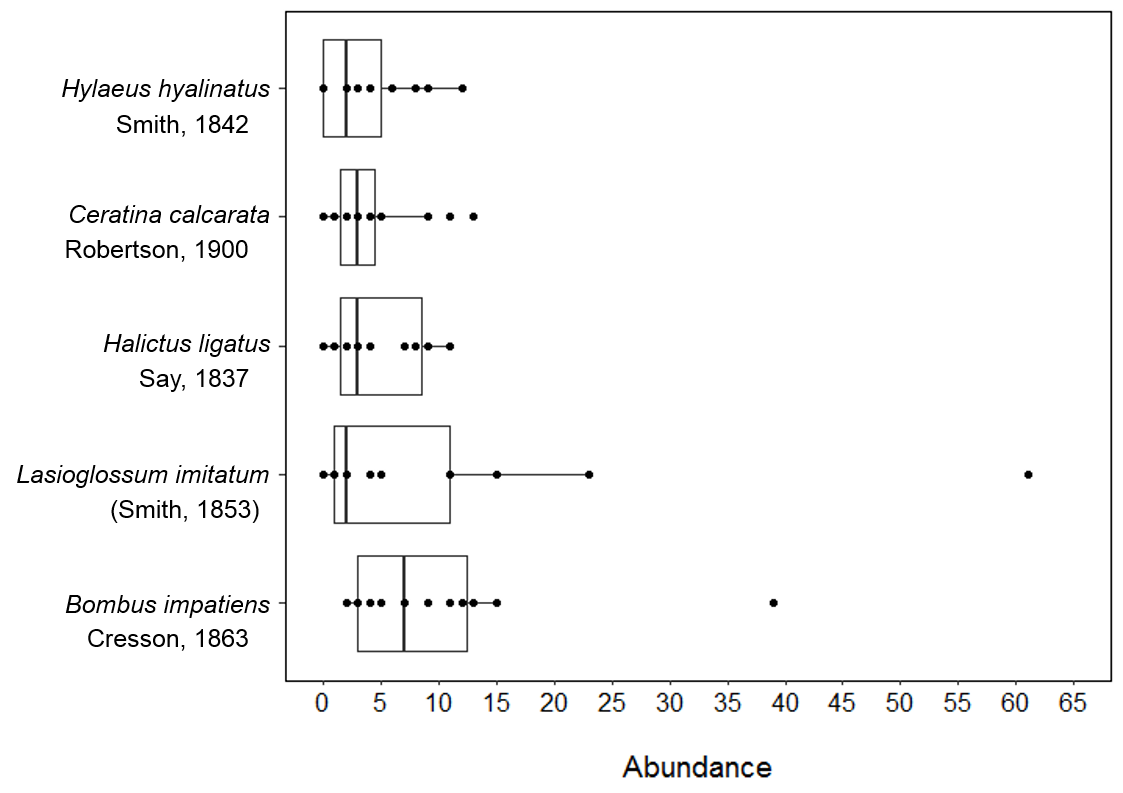


S4 Figure. The most abundant wild bee species collected across sites. *Bombus impatiens* (N = 140), *Lasioglossum imitatum* (N = 139), *Halictus ligatus* (N = 70), *Ceratina calcarata* (N = 60), *Hylaeus hyalinatus* (N = 50)
