# Supplementary material for: The effects of urbanization on bee communities depends on floral resource availability and bee functional traits
Source: PLoS One. 2019 Dec 2;14(12):e0225852. doi: 10.1371/journal.pone.0225852 (PMC6886752; doi:10.1371/journal.pone.0225852)
Supplement: S5 Fig — (DOCX) [file pone.0225852.s005.docx]

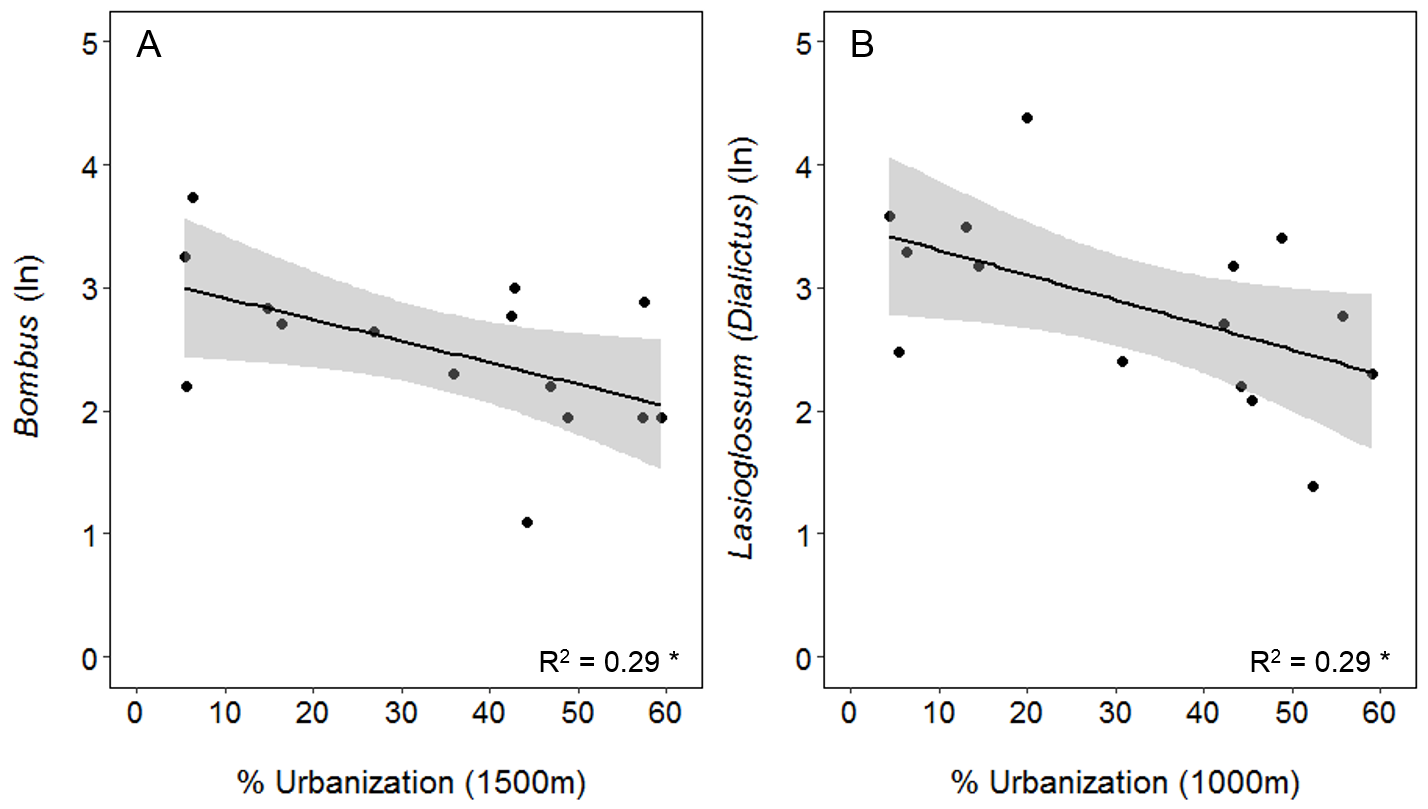


S5 Figure. Effect of urbanization on bee abundance for the eusocial genera (A) *Bombus* and (B) *Lasioglossum (Dialictus)*. The spatial scale that best explained this relationship is shown in parenthesis on the x-axis. Bee abundance data are ln(x+1) transformed. We also found a significant negative relationship between % urbanization and the total number of *Lasioglossum* observed across sites (R^2^=0.53, P=0.0018).
