# Supplementary material for: The effects of urbanization on bee communities depends on floral resource availability and bee functional traits
Source: PLoS One. 2019 Dec 2;14(12):e0225852. doi: 10.1371/journal.pone.0225852 (PMC6886752; doi:10.1371/journal.pone.0225852)
Supplement: S1 Table — (DOCX) [file pone.0225852.s006.docx]

S1 Table. Site-level bee community data. Site numbers correspond with those in Figure S1 and are arranged in order of increasing urbanization at a 1000m radius. Site abbreviations correspond with those in Figure 1 (main text). Species richness and diversity data do not include honey bees. Percent urban is the percent impervious surface surrounding each site within the indicated radius. Bee diversity is the exponential form of the Shannon diversity index.

| Site | Site abbreviation | Farm size (m^2^) | Honey bee abundance | Wild bee abundance | Obs. Spp. richness | Rarefied spp.* | Chao 1 mean | Bee diversity | % Urban (500m) | % Urban (1000m) | % Urban (1500m) | % Urban (2000m) |
| --- | --- | --- | --- | --- | --- | --- | --- | --- | --- | --- | --- | --- |
| 1 | CF | 19,211 | 23 | 61 | 23 | 20.4 | 43.5 | 10.7 | 4.3 | 4.4 | 5.6 | 5.6 |
| 2 | OH | 6,717 | 29 | 76 | 29 | 23.0 | 40.1 | 16.6 | 6.1 | 5.4 | 5.5 | 5.4 |
| 3 | CG | 10,880 | 112 | 167 | 40 | 22.1 | 38.4 | 14.9 | 4.8 | 6.3 | 6.4 | 7.0 |
| 4 | HH | 8,517 | 18 | 88 | 33 | 25.2 | 50.5 | 19.2 | 12.9 | 13.1 | 16.5 | 16.2 |
| 5 | OU | 11,393 | 3 | 68 | 26 | 22.5 | 36.1 | 16.4 | 16.4 | 14.4 | 14.7 | 15.9 |
| 6 | VE | 1,611 | 11 | 212 | 41 | 20.9 | 37.2 | 13.3 | 13.3 | 19.9 | 26.8 | 25.9 |
| 7 | WG | 3,993 | 0 | 51 | 14 | 14.0 | 16.0 | 9.9 | 19.3 | 30.7 | 35.9 | 33.7 |
| 8 | GP | 3,647 | 0 | 58 | 20 | 18.8 | 24.7 | 14.1 | 33.1 | 42.2 | 44.2 | 44.0 |
| 9 | CO | 6,601 | 44 | 119 | 29 | 21.1 | 34.9 | 15.3 | 46.5 | 43.3 | 46.9 | 49.1 |
| 10 | DH | 2,104 | 9 | 108 | 30 | 21.0 | 36.2 | 16.0 | 38.9 | 44.2 | 42.4 | 41.0 |
| 11 | HV | 5,112 | 38 | 82 | 31 | 24.0 | 38.2 | 18.9 | 28.8 | 45.4 | 57.5 | 58.7 |
| 12 | VK | 9,056 | 1 | 163 | 40 | 24.3 | 38.7 | 19.5 | 56.1 | 48.8 | 42.7 | 41.4 |
| 13 | EG | 3,919 | 10 | 60 | 28 | 25.4 | 45.3 | 19.2 | 58.2 | 52.3 | 48.8 | 47.5 |
| 14 | BN | 10,026 | 51 | 104 | 28 | 22.1 | 33.2 | 17.3 | 42.7 | 55.7 | 59.4 | 60.9 |
| 15 | GN | 1,892 | 6 | 72 | 29 | 25.3 | 38.7 | 19.9 | 43.6 | 59.1 | 57.3 | 56.8 |

*Individual based rarefaction was done on the lowest number of recorded specimens across all sites (50 after taxonomic grouping) and is thus a conservative measure of species richness that accounts for differences in specimen number across all sites
