# Supplementary material for: The effects of urbanization on bee communities depends on floral resource availability and bee functional traits
Source: PLoS One. 2019 Dec 2;14(12):e0225852. doi: 10.1371/journal.pone.0225852 (PMC6886752; doi:10.1371/journal.pone.0225852)
Supplement: S2 Table — (DOCX) [file pone.0225852.s007.docx]

S2 Table. Summary of collected bee specimens with functional trait data. Abbreviations are as follows: Native status: N = native, E = exotic; Nesting strategy: GR = ground, ST = stem, C = cavity, SW = soft wood, HW = hard wood, MH = managed hive. Diet Breadth: G = generalist, P = preference (family), SP = specialist (family); Sociality: S = solitary, SB = subsocial, CO = communal, CP = cleptoparasitic, EU = eusocial. Numbers in parenthesis are references. See footnotes for an explanation of symbols used in the table.

| Species | Specimens | Nesting strategy | Diet breadth | Native status | Sociality |
| --- | --- | --- | --- | --- | --- |
| *Agapostemon sericeus* (Förster, 1771) | F: 1 M: 8 | GR (1) | G (2) | N (3) | S (1) |
| *Agapostemon texanus* Cresson, 1872 | F: 4 | GR (1) | G (2) | N (3) | S (1) |
| *Agapostemon virescens* (Fabricus, 1775) | F: 31 M: 8 | GR (4) | G (2) | N (3) | CO (1) |
| *Andrena carlini* Cockerell, 1901 | F: 1 | GR (5) | G (6) | N (6) | S (6) |
| *Andrena commoda* Smith, 1879 | F: 5 | GR (7) | G (6) | N (6) | S (7) |
| *Andrena crataegi* Robertson, 1893 | F: 7 | GR (8) | G (9) | N (9) | CO (8) |
| *Andrena cressonii cressonii* Robertson, 1891 | F: 1 | GR (10) | G (9) | N (9) | S (10) |
| *Andrena geranii* Robertson, 1891 | F: 1 | GR (7) | SP (12)  (Hydrophyllaceae) | N (2) | S (7) |
| *Andrena miserabilis* Cresson, 1872 | F: 1 | GR (13) | G (2) | N (2) | S (7) |
| *Andrena nigrihirta* (Ashmead, 1890) | F: 1 | GR (7) | G (15) | N (15) | S (7) |
| *Andrena platyparia* Robertson, 1895 | M: 4 | GR (7) | P (6)  (Cornaceae) | N (2) | S (7) |
| *Andrena vicina* Smith, 1853 | F: 2 | GR (17) | G (6) | N (6) | S (17) |
| *Andrena wilkella* (Kirby, 1802) | F: 16 M: 14 | GR (18) | P (19)  (Fabaceae) | E (20) | S (18) |
| *Anthidium manicatum manicatum*  (Linnaeus, 1758) | F: 8 M: 19 | C (21) | G (22) | E (22) | S (23) |
| *Anthidium oblongatum oblongatum*  (Illiger, 1806) | F: 9 M: 4 | C (7) | G (22) | E (22) | S (23) |
| *Anthophora terminalis* Cresson, 1869 | F: 1 | ST (7) | G (24) | N (24) | S (25) |
| *Apis mellifera* Linnaeus, 1758 | F: 355 | H (24) | G (26) | E (24) | EU (24) |
| *Augochlora pura pura* (Say, 1837) | F: 15 M: 12 | SW (27) | G (27) | N (2) | S (27) |
| *Augochlorella aurata* (Smith, 1853) | F: 42 M: 2 | GR (28) | G (2) | N (29) | EU (28) |
| *Bombus bimaculatus* Cresson, 1863 | F: 19 M: 19 | GR (30) | G (30) | N (30) | EU (30) |
| #*Bombus fervidus* (Fabricius, 1798) | F: 3 M: 1 | GR (30) | G (30) | N (30) | EU (30) |
| *Bombus griseocollis* (DeGeer, 1773) | F: 9 M: 11 | GR (30) | G (30) | N (30) | EU (30) |
| *Bombus impatiens* Cresson, 1863 | F: 127  M: 13 | GR (30) | G (30) | N (30) | EU (30) |
| *Bombus vagans vagans* Smith, 1854 | F: 3 | GR (30) | G (30) | N (30) | EU (30) |
| *Calliopsis andreniformis* Smith, 1853 | F: 5 M: 12 | GR (31) | G (31) | N (31) | S (31) |
| *Ceratina calcarata* Robertson, 1900 | F: 54 M: 6 | ST (32) | G (24) | N (24) | SB (32) |
| *Ceratina dupla* Say, 1837 | F: 1 | ST (33) | G (24) | N (24) | %SB  (32,33) |
| *Ceratina dupla / mikmaqi* Say, 1837/ Rehan & Sheffield, 2011 | F: 1 | ST (33) | G (24,26) | N (24,34) | %SB  (32,33) |
| *Ceratina mikmaqi* Rehan & Sheffield, 2011 | F: 13 M: 13 | ST (33) | G (26) | N (34) | %SB  (32,33) |
| *Ceratina strenua* Smith, 1879 | F: 7 | ST (35) | G (24) | N (24) | SB (35) |
| *Chelostoma campanularum* (Kirby, 1802) | F: 5 M: 4 | C (36) | SP (36)  (Campanulaceae) | E (37) | S (36) |
| *Chelostoma philadelphi* (Robertson, 1891) | F: 1 | C (38) | SP (12)  (Saxifragaceae) | N (24) | S (38) |
| *Chelostoma rapunculi* (Lepeletier, 1841) | F: 1 M: 2 | C (37) | SP (36)  (Campanulaceae) | E (37) | S (36) |
| *Colletes latitarsis* Robertson, 1891 | F: 2 | GR (40) | PR (12,40)  (Solanaceae) | N (40) | S (40) |
| *Halictus confusus confusus* Smith, 1853 | F: 16 M: 11 | GR (41) | G (2) | N (2) | EU (41) |
| *Halictus ligatus* Say, 1837 | F: 67 M: 3 | GR (3) | G (2) | N (2) | EU (3) |
| *Halictus rubicundus* (Christ, 1791) | F: 8 M: 5 | GR (12) | G (2) | N (2) | EU (3) |
| *Heriades carinata* Cresson, 1864 | F: 18 M: 2 | ST (42,43) | G (24) | N (24) | S (42) |
| *Heriades leavitti* Crawford, 1913 | M: 10 | ST (42,43) | G (24) | N (24) | S (43) |
| *Heriades leavitti / variolosa variolosa*  Crawford, 1913 / (Cresson, 1872) | F: 12 | ST (43,44) | G (24) | N (24,45) | S (43,44) |
| *Hoplitis pilosifrons* (Cresson, 1864) | F: 2 | ST (46) | G (24) | N (24) | S (47) |
| *Hoplitis producta producta* (Cresson, 1864) | F: 6 M: 3 | ST (48) | G (24) | N (24) | S (48) |
| *Hylaeus aff. affinis* (Smith, 1853) | F: 2 | ψC, ST (12,43) | G (2) | N (2) | S (43) |
| *Hylaeus affinis* (Smith, 1853) | F: 2 M: 14 | ψC, ST (12,43) | G (2) | N (2) | S (43) |
| *Hylaeus annulatus* (Linnaeus, 1758) | F: 2 | ψC, %ST (49) | P (50)  (Rosaceae) | N (2) | S (49) |
| *Hylaeus fedorica* (Cockerell, 1909) | F: 7 | ψ %C, ST (7) | G (2) | N (2) | %S (7) |
| *Hylaeus hyalinatus* Smith, 1842 | F: 23 M: 27 | ψ%C, %ST (51) | G (51) | E (51) | %S (7) |
| *Hylaeus illinoisensis* (Robertson, 1896) | M: 1 | ψ%C, ST (7) | G (2) | N (2,51) | %S (7) |
| *Hylaeus leptocephalus* (Morawitz, 1871) | F: 9 M: 12 | ψC, ST, GR (53,54) | P (55)  (Fabaceae) | E (55) | %S (7) |
| *Hylaeus mesillae cressonii* Cockerell, 1896 | F: 16 M: 32 | ψ%C, ST (7,56) | G (2) | N (55) | %S (7) |
| *Hylaeus modestus group(modestus modestus / affinis / illinoisesnis / sp.A)* | F: 43 M: 2 | ψ%C, ST (7) | G (2) | N (2) | %S (7) |
| *Hylaeus modestus modestus* Say, 1837 | F: 1 M: 19 | ψ%C, ST (7) | G (2) | N (2) | %S (7) |
| *Hylaeus near affinis* (Smith, 1853) | F: 1 | ψC, ST (12,43) | G (2) | N (2) | S (43) |
| *Hylaeus spp. 1* | M: 1 | Excluded | Excluded | Excluded | %S (7) |
| *Hylaeus spp. A / Illinoisensis* | M: 4 | ψ%C, ST (7) | G (2) | N (39, 51) | %S (7) |
| *Lasioglossum admirandum* (Sandhouse, 1924) | M: 1 | %GR (7) | G (57) | N (57) | %EU  (12,58,59) |
| *Lasioglossum anomalum* (Robertson, 1892) | F: 31 | %GR (7) | G (57) | N (57) | %EU  (12,58,59) |
| *Lasioglossum bruneri* (Crawford, 1902) | F: 3 | %GR (7) | G (57) | N (57) | %EU  (12,58,59) |
| *Lasioglossum cattallae* (Ellis, 1913) | F: 1 | %GR (7) | G (57) | N (57) | %EU  (12,58,59) |
| *Lasioglossum cinctipes* (Provancher, 1888) | F: 1 | %GR (60) | G (62) | N (61) | EU (63) |
| *Lasioglossum coeruleum* (Robertson, 1893) | F: 3 | SW (64) | G (57) | N (57) | EU (64) |
| *Lasioglossum coriaceum* (Smith, 1853) | F: 2 | %GR (7) | G (65) | N (65) | %S  (59,66) |
| *Lasioglosum cressonii* (Robertson, 1890) | F: 6 | SW (2) | G (57) | N (57) | %EU  (12,58,59) |
| *Lasioglossum ellisiae* (Sandhouse, 1924) | F: 4 M: 1 | %GR (7) | G (57) | N (57) | %EU  (12,58,59) |
| *Lasioglossum ephialtum* Gibbs, 2010 | F: 11 | %GR (7) | G (26) | N (57) | %EU  (12,58,59) |
| *Lasioglossum cf. ephialtum* Gibbs, 2010 | F: 1 | %GR (7) | G (26) | N (57) | %EU  (12,58,59) |
| *Lasioglossum hitchensi* Gibbs, 2012 | F: 25 | %GR (7) | G (57) | N (57) | %EU  (12,58,59) |
| *Lasioglossum illinoense* (Robertson, 1892) | F: 14 | %GR (7) | G (57) | N (57) | %EU  (12,58,59) |
| *Lasioglossum imitatum* (Smith, 1853) | F: 138 M: 1 | GR (67) | G (57) | N (57) | EU (67) |
| *Lasioglossum laevissimum* (Smith, 1853) | F: 1 | GR (60) | G (57) | N (57) | EU (60) |
| *Lasioglossum leucocomum* (Lovell, 1908) | F: 14 M: 2 | %GR (7) | G (57) | N (57) | %EU  (12,58,59) |
| *Lasioglossum leucozonium* (Schrank, 1781) | F: 9 M: 3 | GR (66) | P (65)  (Asteraceae) | E (65) | S (66) |
| *Lasioglossum lineatulum* (Crawford, 1906) | F: 5 M: 2 | GR (69) | G (57) | N (57) | EU (69) |
| *Lasioglossum lionotum* (Sandhouse, 1923) | F: 1 | GR (70) | G (26) | N (57) | CP (71) |
| *Lasioglossum nigroviride* (Graenicher, 1911) | F: 2 | SW (12) | G (57) | N (57) | %EU (12,58,59) |
| *Lasioglossum paradmirandum*  (Kreer & Atwood, 1966) | F: 8 | %GR (7) | G (26) | N (57) | %EU  (12,58,59) |
| *Lasioglossum pectorale* (Smith, 1853) | F: 10 | GR (61) | G (62) | N (61) | S (61) |
| *Lasioglossum perpunctatum* (Ellis, 1913) | F: 1 | %GR (7) | G (57) | N (57) | %EU  (12,58,59) |
| *Lasioglossum pilosum* (Smith, 1853) | F: 21 M: 1 | %GR (7) | G (57) | N (57) | %EU  (12,58,59) |
| *Lasioglossum smilacinae* (Robertson, 1899) | F: 1 | GR  (72) | G (26) | N (57) | %EU  (12,58,59) |
| *Lasioglossum subviridatum* (Cockerell, 1938) | F: 2 | SW (68) | G (26,57) | N (57) | %EU  (12,58,59) |
| *Lasioglossum cf. succipenne* (Ellis, 1913) *sec.* (Gibbs, 2011) | M: 1 | %GR (7) | G (57) | N (57) | %EU  (12,58,59) |
| *Lasioglossum cf. taylorae* Gibbs, 2010 | F: 1 | %GR (7) | %G | N (57) | %EU  (12,58,59) |
| *Lasioglossum tegulare* (Robertson, 1890) | F: 3 | %GR (7) | G (57) | N (57) | %EU  (12,58,59) |
| *&Lasioglossum spp.* | M: 1 | Excluded | %G | Excluded | %EU  (12,58,59) |
| *Lasioglossum versatum* (Robertson, 1902) | F: 9 | GR (73) | G (57) | N (57) | EU (63,73) |
| *Lasioglossum vierecki* (Crawford, 1904) | F: 10 M: 1 | GR (74) | G (57) | N (57) | S (74,75) |
| *Lasioglossum weemsi* (Mitchell, 1960) | F: 8 | %GR (7) | G (26) | N (57) | %EU  (12,58,59) |
| *Lasioglossum zephyrum* (Smith, 1853) | F: 1 | GR (76) | G (57) | N (57) | EU (76) |
| *Lasioglossum zonulum* (Smith, 1848) | F: 1 | GR (66) | G (65) | E (77) | S (66) |
| *Megachile campanulae* (Robertson, 1903) | F: 6 M: 5 | ΨC, ST (43,78) | G (24) | N (24,78) | S (12) |
| *Megachile centuncularis* (Linnaeus, 1758) | F: 1 M: 1 | ΨC, ST (43,79) | G (24) | N (24,78) | S (12) |
| *Megachile mendica* Cresson, 1878 | M: 4 | ΨC, ST, GR (12) | G (24) | N (24,78) | S (12) |
| *Megachile montivaga* Cresson, 1878 | F: 1 | +ST, GR (78) | G (24) | N (24,78) | S (12) |
| *Megachile nr. relativa* Cresson, 1878 | F: 1 | ΨC, ST (79,80) | G (24) | N (24,78) | S (12) |
| *Megachile pugnata pugnata* Say, 1837 | F: 1 | ΨC, ST, SW (78) | SP (81,82)  (Asteraceae) | N (24,78) | S (12) |
| *Megachile rotundata* (Fabricius, 1787) | F: 6  M: 12 | ΨC, GR (78) | G (24) | E (78) | S (83) |
| *Megachile texana* Cresson, 1878 | F: 1  M: 1 | GR (78) | G (24) | N (78) | S (12) |
| *Melissodes agilis* Cresson, 1878 | F: 10  M: 21 | GR (84) | SP (12,84)  (Asteraceae) | N (84) | S (84) |
| *Melissodes bimaculatus bimaculatus*  (Lepeletier, 1825) | F: 26  M: 8 | GR (85) | G (86) | N (86) | S (85) |
| *Melissodes desponsus* (Smith, 1854) | F: 1  M: 3 | %GR (87) | SP (12,87)  (Asteraceae) | N (87) | %S (87) |
| *Melissodes subillatus* LaBerge, 1961 | F: 15  M: 3 | %GR (84) | SP (84)  (Asteraceae) | N (84) | %S (84) |
| *Nomada denticulata* Robertson, 1902 | M: 1 | GR (12) | G (24) | N (24) | CP (24) |
| *Nomada maculata* Cresson, 1863 | F: 1 | GR (12) | G (24) | N (24) | CP (24) |
| *Nomada pygmaea* Cresson, 1863 | F: 1 | GR (12) | G (24,26) | N (24) | CP (24) |
| *Osmia albiventris* Cresson, 1864 | F: 1 | C (88) | G (24) | N (24) | S (89) |
| *Osmia atriventris* Cresson, 1864 | F: 1 | ΨC, ST (88) | G (24) | N (24) | S (90) |
| *Osmia caerulescens* (Linnaeus, 1758) | F: 1 | C (89) | G (24,26) | E (12) | S (89) |
| *Osmia conjuncta* Cresson, 1864 | F: 11 | C (91) | G (24) | N (24) | %S (91) |
| *Osmia cornifrons* (Radoszkowski, 1887) | F: 1 | C (92) | G (24) | E (12) | S (92) |
| *Osmia pumila* Cresson, 1864 | F: 5  M: 1 | ΨC, ST (88) | G (24) | N (24) | S  (94) |
| *Peponapis pruinosa* (Say, 1837) | F: 9 M: 13 | GR (95) | SP (96) (Cucurbitaceae) | N (96) | S (95) |
| *Sphecodes cressonii* (Robertson, 1903) | F: 1 | (2) | G (2) | N (2) | CP (2) |
| *Sphecodes dichrous* Smith, 1853 | M: 2 | (2) | G (2) | N (2) | CP (2) |
| *Stelis lateralis* Cresson, 1864 | F: 1 M: 2 | ST (12) | G (24) | N (24) | CP (24) |
| *Stelis louisae* Cockerell, 1911 | M: 4 | ST (97,98) | G (24) | N (24) | CP (24) |
| *Triepeolus remigatus* (Fabricius, 1804) | F: 2 | GR (99) | G (99) | N (99) | CP (99) |
| *Xylocopa virginica virginica* (Linnaeus, 1771) | F: 9 M: 2 | HW (100) | G (24) | N (24) | +SB, S (100) |

# One male specimen of *Bombus fervidus* (Cresson) was identified from photographs by Rob Jean (Environmental Solutions and Innovations).

% traits that were inferred based on similar species and from the listed sources.

@ *Lasioglossum, Hylaeus,* and *Ceratina* specimens without average body size measurements were included in counts of small bees for analysis because all members of these groups have average body sizes less than 10mm.

Ψ *Hylaeus* and *Megachile* that nested in stems and cavities were listed as cavity nesters in final analysis. All *Hylaeus* were assumed to nest in both substrates.

& This *Lasioglossum (Dialictus)* specimen was too damaged to be accurately identified to species.

+ Species that had been recorded nesting in multiple substrates or at different levels of sociality were sorted into the substrate they were most commonly recorded in. This substrate that species were sorted into is indicated with “+”

**References**

1. Eickwort GC. Aspects of the Nesting Biology of Five Nearctic Species of Agapostemon (Hymenoptera : Halictidae). J Kansas Entomol Soc. 1981;54(2):337–51.

2. Mitchell TB. Bees of the eastern United States. I. Technical bulletin (North Carolina Agricultural Experiment Station); 1960. 538 p.

3. Roberts RB. Bees of Northwestern America: Agapostemon (Hymenoptera: Halictidae). Corvallis: Agricultural Experiment Station. 1973.

4. Abrams J, Eickwort GC. Biology of the communal sweat bee Agapostemon virescens (Hymenoptera: Halictidae) in New York State. Search Agric. 1980;1:1–20.

5. Schrader MN, LaBerge WE. The Nesting Biology of the Bees Andrena (Melandrena) regularis Malloch and Andrena (Melandrena) carlini Cockerell (Hymenoptera: Andrenidae). Illinois Nat Hist Surv. 1978;108:3–24.

6. Bouseman JK, LaBerge WE. A revision of the bees of the genus Andrena of the Western Hemisphere. Part IX. Subgenus Melandrena. Trans Am Entomol Soc. 1978;104(3):275–389.

7. Michener CD. The bees of the world [Internet]. 2nd ed. Vol. 85, The Johns Hopkins University Press. Baltimore, Maryland: The Johns Hopkins University Press; 2007. 963 p. Available from: http://books.google.com/books?id=bu_1gmY13FIC

8. Osgood EA. Biology of Andrena crataegi Robertson (Hymenoptera : Andrenidae), a Communally Nesting Bee. J New York Entomol Soc. 1989;97(1):56–64.

9. LaBerge WE. A revision of the bees of the genus Andrena of the Western Hemisphere. Part II. Plastandrena, Aporandrena, Charitandrena. Trans Am Entomol Soc. 1969;95(1):1–47.

10. Miliczky ER. Observations on the Bionomics of the Bee Andrena (Tylandrena) erythrogaster Ashmead (Hymenoptera: Andrenidae). Illinois Nat Hist Surv. 1988;130:502–9.

11. LaBerge WE. A revision of the bees of the genus Andrena of the western hemisphere. Part XI. Minor subgenera and subgeneric key. Trans Am Entomol Soc. 1985;111(4):441–567.

12. Gibbs J, Ascher JS, Rightmyer MG, Isaacs R. The bees of Michigan (Hymenoptera: Apoidea: Anthophila), with notes on distribution, taxonomy, pollination, and natural history. Vol. 4352, Zootaxa. 2017. 1-160 p.

13. Norden BB, Scarbrough AG. Nesting biology of Andrena (Larandrena) miserabilis Cresson and description of the prepupa (Hymenoptera: Andrenidae). Brimleyana. 1979;2:141–6.

14. Ribble DW. The Monotypic North American Subgenus" Larandrena" of" Andrena"(Hymenoptera: Apoidea). 1967.

15. Laberge WE, Ribble DW. A revision of the bees of the genus Andrena of the western hemisphere. Part VII. Subgenus Euandrena. Trans Am Entomol Soc. 1975;101(3):371–446.

16. LaBerge WE, Ribble DW. A revision of the bees of the genus Andrena of the western hemisphere. Part V. Gonandrena, Geissandrena, Parandrena, Pelicandrena. Trans Am Entomol Soc. 1972;98(3):271–358.

17. Miliczky ER, Osgood EA. Bionomics of Andrena (Melandrena) vicina Smith in Maine and Washington, with new parasite records for A.(M.) regularis Malloch and a review of Melandrena biology. J Kansas Entomol Soc. 1995;68(1).

18. Atwood CE. Studies on the apoidea of western nova scotia with special reference to visitors to apple bloom. Can J Res. 1933;9(5):443–57.

19. LaBerge WE. A revision of the bees of the genus Andrena of the western hemisphere. Part XIII. Subgenera Simandrena and Taeniandrena. Trans Am Entomol Soc. 1989;115(1):1–56.

20. Malloch JR. Occurrence of a European solitary bee (Andrena wilkella Kirby) in the eastern United States. Proc Biol Soc Washingt. 1918;31:61–4.

21. Gibbs J, Sheffield CS. Rapid Range Expansion of the Wool-Carder Bee , Anthidium manicatum (Linnaeus) (Hymenoptera : Megachilidae), in North America. 2009;82(1):21–9.

22. Gonzalez VH, Griswold TL. Wool carder bees of the genus Anthidium in the Western Hemisphere (Hymenoptera: Megachilidae): Diversity, host plant associations, phylogeny, and biogeography. Zool J Linn Soc. 2013;168(2):221–425.

23. Burks BD. The Pollen-Collecting Bees of The Anthidiini of California (Hymenoptera: Megachilidae). Bull Entomol Soc Am. 1968;14(3):256–7.

24. Mitchell TB. Bees of the eastern United States. II. Technical bulletin (North Carolina Agricultural Experiment Station); 1962. 557 p.

25. Medler JT. Anthophora (Clisodon) terminalis Cresson in trap-nests in Wisconsin (Hymenoptera: Anthophoridae). Can Entomol. 1964;96(10):1332–6.

26. Ascher JS, Pickering J. Discover Life bee species guide and world checklist (Hymenoptera: Apoidea: Anthophila) [Internet]. 2018. Available from: http://www.discoverlife.org/mp/20q?guide=Apoidea_species

27. Stockhammer KA. Nesting habits and life cycle of a sweat bee, Augochlora pura (Hymenoptera: Halictidae). J Kansas Entomol Soc. 1966;39(2):157–92.

28. Mueller UG. Life History and Social Evolution of the Primitively Eusocial Bee Augochlorella striata (Hymenoptera : Halictidae). J Kansas Entomol Soc. 1996;69(4):116–38.

29. Ordway E. ystematics of the genus Augochlorella (Hymenoptera, Halictidae) North of Mexico. Univ Kansas Sci Bull. 1966;46(16):509–624.

30. Williams P, Thorp R, Richardson L, Colla SR. Bumble bees of North America. Princeton, NJ: Princeton University Press; 2014. 208 p.

31. Shinn AF. A revision of the bee genus Calliopsis and the biology and ecology of C. andreniformis (Hymenoptera, Andrenidae). Univ Kansas Sci Bull. 1967;46:753–936.

32. Rehan SM, Richards MH. Nesting biology and subsociality in ceratina calcarata (Hymenoptera: Apidae). Can Entomol. 2010;142(1):65–74.

33. Vickruck JL, Rehan SM, Sheffield CS, Richards MH. Nesting biology and DNA barcode analysis of ceratina dupla and C. mikmaqi, and comparisons with C. calcarata (Hymenoptera: Apidae: Xylocopinae). Can Entomol. 2011;143(3):254–62.

34. Rehan SM, Sheffield CS. Morphological and molecular delineation of a new species in the Ceratina dupla species-group (Hymenoptera: Apidae: Xylocopinae) of eastern North America. Zootaxa. 2011;2873:35–50.

35. Kislow CJ. The Comparative Biology of two Species of Small Carpenter Bees Ceratina Strenua F. Smith and C. Calcarata Robertson (Hymenoptera, Xylocopinae). University of Georgia; 1976.

36. Käpylä M. Bionomics of five wood-nesting solitary species of bees (Hym., Megachilidae), with emphasis on flower relationships. Biol Rev Rep Univ Jyväskylä. 1978;5:3–89.

37. Eickwort GC. Two European Species of Chelostoma Established In New York State (Hymenoptera: Megachilidae). Psyche (Stuttg). 1980;87(3–4):315–23.

38. Krombein K V. Biological notes on Prochelostoma philadelphi (Robertson) (Hymenoptera, Megachilidae). Entomol News. 1959;70:135–6.

39. Buck M, Paiero SM, Marshall SA. New Records of Native and Introduced Aculeate Hymenoptera From Ontario , With Keys To Eastern Canadian Species of Cerceris ( Crabronidae ) and Eastern Nearctic Species of Chelostoma ( Megachilidae ). J Entomol Soc Ontario. 2005;136:37–52.

40. Stephen WP. A Revision of the Bee Genus Colletes in America North of Mexico (Hymenoptera, Colletidae). Univ Kansas Sci Bull. 1954;36:149–527.

41. Richards MH, Vickruck JL, Rehan SM. Colony social organisation of Halictus confusus in Southern Ontario, with comments on sociality in the subgenus H. (Seladonia). J Hymenopt Res. 2010;19:144–58.

42. Matthews RW. The biology of Heriades carinata Cresson (Hymenoptera, Megachilidae). Contrib Am Entomol Inst. 1965;1:1–33.

43. Krombein KV. Trap-nesting wasps and bees: Life histories, nests, and associates. Washington, D.C.: Smithsonian Institution Press; 1967. 570 p.

44. Fischer RL. A nest of Heriades variolosus (Cress.);(Hymenoptera: Megachilidae). Can Entomol. 1955;87(1):33–6.

45. Hurd PD, Michener CD. The megachiline bees of California (Hymenoptera: Megachilidae) (Vol. 3). University of California Press; 1955.

46. Michener CD. Some biological observations on Hoplitis pilosifrons and Stelis lateralis (Hymenoptera, Megachilidae). J Kansas Entomol Soc. 1955;28(3):81–7.

47. Michener CD. Some biological observations on Hoplitis pilosifrons and Stelis lateralis (Hymenoptera, Megachilidae). J Kansas Entomol Soc. 1955;28(3):81–7.

48. Medler JT. A note on Hoplitis producta (Cress.) in Wisconsin (Hymenoptera: Megachilidae). Can Entomol. 1961;93(7):571–3.

49. Scott VL. The Great Lakes Entomologist Phenology and Trap Selection of Three Species of Hylaeus (Hymenoptera: Colletidae) in Upper Michigan. Gt Lakes Entomol [Internet]. 1995;27(1). Available from: http://scholar.valpo.edu/tgle%0Ahttp://scholar.valpo.edu/tgle/vol27/iss1/5

50. Scott V. Pollen Selection by Three Species of Hylaeus in Michigan (Hymenoptera: Colletidae). J Kansas Entomol Soc [Internet]. 1996;69(4):195–200. Available from: http://www.jstor.org/stable/25085717

51. Ascher JS. Hylaeus hyalinatus Smith, a European bee new to North America, with notes on other adventive bees (Hymenoptera: Apoidea). Proc Entomol Soc Washingt. 2001;103(1):184–90.

52. Dathe HH. Arten der gattung Hylaeus F. in Europa (Hymenoptera: Apoidea, Colletidae). Mitteilungen aus dem Zool Museum Berlin. 1980;

53. Torchio PF. The nesting biology of Hylaeus bisinuatus Forster and development of its immature forms (Hymenoptera: Colletidae). J Kansas Entomol Soc. 1984;276–97.

54. Barrows EM. Occupancy by Hylaeus of Subterranean Halictid Nests (Hymenoptera: Apoidea). Psyche A J Entomol. 1975;82(1):74–7.

55. Snelling RR. Studies on North American Bees of the Genus Hylaeus. 5. The Subgenera Hylaeus, S. Str. And Paraprosopis (Hymenoptera: Colletidae). Los Angeles Cty Museum Contrib Sci. 1970;180:1–59.

56. Hicks CH. Nesting habits and parasites of certain bees of Boulder County, Colorado. 1926.

57. Gibbs J. Revision of the metallic species of Lasioglossum (Dialictus) in Canada (Hymenoptera, Halictidae, Halictini). Vol. 382, Zootaxa. 2010. 1-382 p.

58. Danforth BN, Conway L, Ji S. Phylogeny of eusocial Lasioglossum reveals multiple losses of eusociality within a primitively eusocial clade of bees (Hymenoptera: Halictidae). Syst Biol. 2003;52(1):23–36.

59. Gibbs J, Brady SG, Kanda K, Danforth BN. Phylogeny of halictine bees supports a shared origin of eusociality for Halictus and Lasioglossum (Apoidea: Anthophila: Halictidae). Mol Phylogenet Evol [Internet]. 2012;65(3):926–39. Available from: http://dx.doi.org/10.1016/j.ympev.2012.08.013

60. Packer L, Jessome V, Lockerbie C, Sampson B. The phenology and social biology of four sweat bees in a marginal environment: Cape Breton Island. Can J Zool. 1989;67(12):2871–7.

61. Gibbs J, Packer L, Dumesh S, Danforth BN. Revision and reclassification of Lasioglossum (Evylaeus), L. (Hemihalictus) and L. (Sphecodogastra) in eastern North America (Hymenoptera: Apoidea: Halictidae). Zootaxa. 2013;3672(1):001-117.

62. Moure JS, Hurd Jr PD. An annotated catalog of the halictid bees of the western hemisphere (Hymenoptera: Halictidae). An annotated catalog of the halictid bees of the Western Hemisphere (Hymenoptera: Halictidae). Washington, D.C.: Smithsonian Inst.; 1987. 405 p.

63. Knerer G, Atwood CE. Polymorphism in some Nearctic halictine bees. Science (80- ). 1966;152(3726):1262–3.

64. Stockhammer KA. Some Notes on the Biology of the Blue Sweat Bee , Lasioglossum coeruleum (Apoidea : Halictidae). J Kansas Entomol Soc. 1967;40(2):177–89.

65. McGinley RL. Studies of Halictinae (Apoidea: Halictidae), I: revision of new world Lasioglossum curtis. Smithson Contrib to Zool. 1986;429:294.

66. Pesenko YA, Banaszak J, Radchenko VG, Cierzniak T. Bees of the family Halictidae (excluding Sphecodes) of Poland: taxonomy, ecology, bionomics. Bydgszcz: Wyzszej Szkoly Pedagogicznej; 2000. 348 p.

67. Michener CD, Wille A. The Bionomics of a Primitively Social Bee Lasioglossum inconspicuum. Univ Kansas Sci Bull. 1961;42(11):1123–202.

68. Gibbs J. Revision of the metallic *Lasioglossum* (*Dialictus*) of eastern North America (Hymenoptera: Halictidae: Halictini). Vol. 216, Zootaxa. 2011. 1-216 p.

69. Eickwort GC. First Steps into Eusociality : The Sweat Bee Dialictus lineatulus. Florida Entomol [Internet]. 1986;69(4):742–54. Available from: http://www.jstor.org/stable/3495222

70. Wcislo WT. Invasion of nests of Lasioglossum imitatum by a social parasite, Paralictus asteris (Hymenoptera: Halictidae). Ethology. 1997;103(1):1–11.

71. Wcislo WT. Invasion of nests of Lasioglossum imitatum by a social parasite, Paralictus asteris (Hymenoptera: Halictidae). Ethology. 1997;103(1):1–11.

72. Brittain WH. Apple pollination studies in the Annapolis Valley, N.S. Canada. Dom Canada Dep Agric Bull. 1933;162:1–198.

73. Breed MD. Life Cycle and Behavior of a Primitively Social Bee , Lasioglossum rohweri ( Hymenoptera : Halictidae ) Author ( s ): Michael D . Breed Source : Journal of the Kansas Entomological Society , Vol . 48 , No . 1 ( Jan ., 1975 ), pp . 64-80 Published by : Kan. J Kansas Entomol Soc. 1975;48(1):64–80.

74. Knerer G. Synergisitc evolution of halictine nest architecture and social behavior. Can J Zool. 1969;47(5):925–30.

75. Packer L. Multiple-foundress associations in sweat bees. In: Queen number and sociality in insects. New York, New York: Oxford University Press; 1993. p. 309–313.

76. Batra SWT. Behavior of the social bee, Lasioglossum zephyrum, within the nest (Hymenoptera: Halictidae). Insectes Soc. 1964;11:159–86.

77. Giles V, Ascher JS. A survey of the bees of the Black Rock Forest Preserve, New York (Hymenoptera: Apoidea). J Hymenopt Res. 2006;15(2):208–31.

78. Sheffield CS, Ratti C, Packer L, Griswold T. Leafcutter and Mason Bees of the Genus Megachile Latreille (Hymenoptera: Megachilidae) in Canada and Alaska. Can J Arthropod Identif [Internet]. 2011;18(18):1–107. Available from: http://biologicalsurvey.ca/ejournal/srpg_18/srpg_18.html

79. Sheffield CS, Kevan PG, Westby SM, Smith RF. Diversity of cavity-nesting bees ( Hymenoptera : Apoidea ) within apple orchards and wild habitats in the Annapolis Valley , Nova Scotia , Canada Diversity of cavity-nesting bees ( Hymenoptera : Apoidea ) within apple orchards and wild habitats in the Ann. Can Entomol. 2008;140(2):235–49.

80. Medler JT, Koeorber TW. Biology of Megachile relativa Cresson (Hymenoptera, Megachilidae) in trap-nests in Wisconsin. Ann Entomol Soc Am. 1958;51(4):337–44.

81. Tepedino VJ, Frohlich DR. Mortality factors, pollen utilization, and sex ratio in Megachile pugnata Say (Hymenoptera: Megachilidae), a candidate for commercial sunflower pollination. J New York Entomol Soc. 1982;269–74.

82. Parker FD, Frohlich DR. Hybrid sunflower pollination by a manageable composite specialist: the sunflower leafcutter bee (Hymenoptera: Megachilidae). Environ Entomol. 1983;12(2):576–81.

83. Pitts-Singer TL, Cane JH. The Alfalfa Leafcutting Bee, *Megachile rotundata* : The World’s Most Intensively Managed Solitary Bee. Annu Rev Entomol [Internet]. 2011;56(1):221–37. Available from: http://www.annualreviews.org/doi/10.1146/annurev-ento-120709-144836

84. LaBerge WE. A revision of the bees of the genus Melissodes in North and Central America. Part III (Hymenoptera, Apidae). Univ Kansas Sci Bull. 1961;42:283–663.

85. Ashmead WH. The Habits of the Aculeate Hymenoptera.—I. Psyche (Stuttg). 1894;7(19):19–26.

86. LaBerge WE. A revision of the bees of the genus Melissodes in North and Central America. Part I. Univ Kansas Sci Bull. 1956;37:911–1194.

87. LaBerge WE. A revision of the bees of the genus Melissodes in North and Central America. Part II (Hymenoptera, Apidae). Univ Kansas Sci Bull. 1956;38:533–78.

88. Cane JH, Griswold T, Parker FD. Substrates and Materials Used for Nesting by North American Osmia Bees (Hymenoptera: Apiformes: Megachilidae). Ann Entomol Soc Am [Internet]. 2007;100(3):350–8. Available from: https://academic.oup.com/aesa/article/100/3/350-358/8333

89. Medler JT. Biology of Osmia in trap nests in Wisconsin (Hymenoptera: Megachilidae). Ann Entomol Soc Am. 1967;60(2):338–44.

90. Fye RE. Biology of Apoidea Taken in Trap Nests in Northwestern Ontario (Hymenoptera). Can Entomol. 1965;97(8):863–77.

91. Rau P. The life-history of Osmia lignaria and O. cordata, with notes on O. conjuncta. Ann Entomol Soc Am. 1937;30(2):324–43.

92. Batra SWT. Management of hornfaced bees for orchard pollination [Internet]. 2007 [cited 2018 Apr 10]. Available from: http://www.pollinatorparadise.com/solitary_bees/hornface.htm

93. Yasumatsu K, Hirashima Y. Revision of the genus Osmia of Japan and Korea (Hymenoptera: Megachilidae). Mushi , Fukuoka. 1950;21:1–18.

94. Goodell K. Food availability affects Osmia pumila (Hymenoptera: Megachilidae) foraging, reproduction, and brood parasitism. Oecologia. 2003;134(4):518–27.

95. Hurd PD, Linsley EG, Michelbacher a. D. Ecology of the squash and gourd bee, Peponapis pruinosa, on cultivated cucurbits in California (Hymenoptera: Apoidea). Smithson Contrib to Zool. 1974;168:1–17.

96. Hurd PD, Linsley EG. The squash and gourd bees- genera Peponapis Robertson and Xenoglossa Smith- inhabiting America north of Mexico (Hymenoptera: Apoidea). Hilgardia. 1964;35(15):375–477.

97. Baker JR, Kuhn ED, Bambara SB. Nests and immature stages of leafcutter bees (Hymenoptera: Megachilidae). J Kansas Entomol Soc. 1985;58(2):290–313.

98. Parker FD, Cane JH, Frankie GW, Vinson SB. Host records and nest entry by Dolichostelis, a kleptoparasitic anthidiine bee (Hymenoptera: Megachilidae). Registros de hospederos y entrada al nido por parte de Dolichostelis, una abeja clepotoparásita (Hymenoptera: Megachilidae). Pan-Pac Entomol. 1987;63(2):172–7.

99. Rightmyer MG. A review of the cleptoparasitic bee genus Triepeolus (Hymenoptera: Apidae).-Part I. Zootaxa. 2008;1710:1–170.

100. Richards MH. Colony Social Organisation and Alternative Social Strategies in the Eastern Carpenter Bee, Xylocopa virginica. J Insect Behav. 2011;24(5):399–411.
