# Supplementary material for: The effects of urbanization on bee communities depends on floral resource availability and bee functional traits
Source: PLoS One. 2019 Dec 2;14(12):e0225852. doi: 10.1371/journal.pone.0225852 (PMC6886752; doi:10.1371/journal.pone.0225852)
Supplement: S3 Table — (DOCX) [file pone.0225852.s008.docx]

S3 Table. The species number and bloom cover for all surveyed plants, listed by A) geographic origin and B) plant category.

| Category | Species number | Bloom cover (m^2^) |
| --- | --- | --- |
| A. Geographic origin |  |  |
| Native | 86 | 58.435 |
| Exotic | 195 | 116.071 |
| B. Category |  |  |
| Noxious weed | 7 | 19.873 |
| Ornamental & crop | 119 | 60.723 |
| Wildflower | 159 | 93.584 |
| Total | 285 | 174.619 |
