# Supplementary material for: The effects of urbanization on bee communities depends on floral resource availability and bee functional traits
Source: PLoS One. 2019 Dec 2;14(12):e0225852. doi: 10.1371/journal.pone.0225852 (PMC6886752; doi:10.1371/journal.pone.0225852)
Supplement: S4 Table — (DOCX) [file pone.0225852.s009.docx]

S4 Table. Summary of AICc values for model selection. Final models used for analysis of bee response variables are in bold.

| **Response Variable** | **Parameters** | AICc | ΔAICc |
| --- | --- | --- | --- |
| *A. Community level* |  |  |  |
| **Total bee abundance** | 1500m | 24.10 | 0 |
| Urbanization predictor | 1000m | 24.10 | 0 |
|  | 2000m | 24.11 | 0.01 |
|  | 500m | 24.14 | 0.04 |
| Floral resource predictor | 1500m + bloom cover | 25.15 | 0 |
|  | 1500m + plant spp. richness | 26.83 | 1.68 |
|  | 1500m + plant spp. richness + bloom cover | 29.69 | 4.54 |
| Farm size | 1500m + bloom cover | 25.15 | 0 |
|  | 1500m + bloom cover + farm size | 27.44 | 2.3 |
| Temperature | **1500m + bloom cover** | **25.15** | **0** |
|  | 1500m + bloom cover + minimum temperature | 26.91 | 1.77 |
| **Bee species richness** | 500m | -10.40 | 0 |
| Urbanization predictor | 1000m | -9.66 | 0.75 |
|  | 2000m | -9.47 | 0.93 |
|  | 1500m | -9.42 | 0.98 |
| Floral resource predictor | 500m + plant spp. richness | -12.22 | 0 |
|  | 500m + plant spp. + bloom cover | -7.60 | 4.61 |
|  | 500m + bloom cover | -7.24 | 4.98 |
| Farm size | 500m + plant spp. richness | -12.22 | 0 |
|  | 500m + plant spp. richness + farm size | -7.86 | 4.36 |
| Temperature | **500m + plant spp.** **richness** | **-10.07** | **0** |
|  | 500m + plant spp. richness + minimum temperature | -5.49 | 4.58 |
| **Bee evenness** | 1000m | -75.23 | 0 |
| Urbanization predictor | 500 m | -74.66 | 0.57 |
|  | 2000m | -74.10 | 1.12 |
|  | 1500m | -74.07 | 1.16 |
| Floral resource predictor | 1000m + plant spp. richness | -72.60 | 0 |
|  | 1000m + bloom cover | -72.56 | 0.04 |
|  | 1000m + plant spp. richness + bloom cover | -71.51 | 1.09 |
| Farm size | 1000m + plant spp. richness | -72.60 | 0 |
|  | 1500m + plant spp. richness + farm size | -69.49 | 3.11 |
| Temperature | **1000m + plant spp. richness** | -72.60 | 0 |
|  | 1000m + plant spp. richness + minimum temperature | -67.93 | 4.66 |
| **Bee diversity** | 500m | -3.28 | 0 |
|  | 1000m | -1.97 | 1.31 |
|  | 2000m | -1.19 | 2.09 |
|  | 1500m | -1.04 | 2.24 |
| Floral resource predictor | 500m + plant spp. richness | -4.80 | 0 |
|  | 500m + plant spp. richness + bloom cover | -1.85 | 2.94 |
|  | 500m + bloom cover | 0.53 | 5.33 |
| Farm size | 500m + plant spp. richness | -4.80 | 0 |
|  | 500m + plant spp. richness + farm size | 0.25 | 4.55 |
| Temperature | **500m + plant spp. richness** | **-4.80** | **0** |
|  | 500m + plant spp. richness + minimum temperature | -0.41 | 4.39 |
| *B. Functional groups* |  |  |  |
| **Native bees** | 1000m | 23.18 | 0 |
| Urbanization predictor | 1500m | 23.39 | 0.21 |
|  | 500m | 23.43 | 0.25 |
|  | 2000m | 23.45 | 0.27 |
| Floral resource predictor | 1000m + bloom cover | 24.80 | 0 |
|  | 1000m + plant spp. richness | 26.08 | 1.28 |
|  | 1000m + plant spp. richness + bloom cover | 29.33 | 4.53 |
| Farm Size | 1000m + bloom cover | 24.80 | 0 |
|  | 1000m + bloom cover + farm size | 27.25 | 2.46 |
| Temperature | **1000m + bloom cover** | **24.80** | **0** |
|  | 1000m + bloom cover + minimum temperature | 25.63 | 0.83 |
| **Exotic Bees** | 500m | 34.88 | 0 |
| Urbanization predictor | 1000m | 39.32 | 4.44 |
|  | 2000m | 41.33 | 6.45 |
|  | 1500m | 41.40 | 6.52 |
| Floral resource predictor | 500m + bloom cover | 33.68 | 0 |
|  | 500m + plant spp. | 37.87 | 4.19 |
|  | 500m + plant spp. + bloom cover | 38.34 | 4.67 |
| Farm size | 500m + bloom cover | 33.68 | 0 |
|  | 500m + bloom cover + farm size | 37.35 | 3.67 |
| Temperature | **500m + bloom cover** | **33.68** | **0** |
|  | 500m + bloom cover + minimum temperature | 37.94 | 4.27 |
| **Ground-nesting bees** | 1000m | 21.44 | 0 |
| Urbanization predictor | 2000m | 21.57 | 0.13 |
|  | 1500m | 21.64 | 0.20 |
|  | 500m | 23.70 | 2.27 |
| Floral resource predictor | 1000m + total bloom | 22.55 | 0 |
|  | 1000m + plant spp. richness | 24.89 | 2.35 |
|  | 1000m + plant spp. richness + total bloom | 27.20 | 4.65 |
| Farm size | 1000m + total bloom | 22.55 | 0 |
|  | 1000m + total bloom + farm size | 22.65 | 0.10 |
| Temperature | **1000m + total bloom** | **22.55** | **0** |
|  | 1000m + total bloom + minimum temperature | 26.68 | 4.14 |
| **Above-ground nesting bees** | 500m | 43.03 | 0 |
| Urbanization predictor | 1000m | 43.77 | 0.74 |
|  | 2000m | 43.96 | 0.74 |
|  | 1500m | 44.20 | 1.17 |
|  | 500m + plant spp. richness | 44.70 | 0 |
|  | 500m + total bloom | 45.56 | 0.86 |
|  | 500m + plant spp. richness + total bloom | 49.04 | 4.34 |
| Farm size | 500m + plant spp. richness | 44.70 | 0 |
|  | 500m + plant spp. richness + farm size | 48.84 | 4.13 |
| Temperature | **500m + plant spp. richness** | **44.70** | **0** |
|  | 500m + plant spp. richness + minimum temperature | 46.27 | 1.57 |
| **Solitary bees** | 1500m | 32.18 | 0 |
| Urbanization predictor | 1000m | 32.19 | 0.01 |
|  | 2000m | 32.28 | 0.10 |
|  | 500m | 32.51 | 0.33 |
| Floral resource predictor | 1500m + total bloom | 35.64 | 0 |
|  | 1500m + plant spp. richness | 35.90 | 0.27 |
|  | 1500m + plant spp. richness + total bloom | 40.30 | 4.67 |
| Farm size | 1500m + total bloom | 35.64 | 0 |
|  | 1500m + total bloom + farm size | 36.38 | 0.74 |
| Temperature | **1500m + total bloom** | **35.64** | **0** |
|  | 1500m + total bloom + minimum temperature | 37.41 | 1.77 |
| **Eusocial bees** | 1000m | 23.10 | 0 |
| Urbanization predictor | 1500m | 23.27 | 0.17 |
|  | 2000m | 23.66 | 0.55 |
|  | 500m | 26.55 | 3.45 |
| Floral resource predictor | 1000m + total bloom | 21.37 | 0 |
|  | 1000m + plant spp. richness | 24.00 | 2.63 |
|  | 1500m + plant spp. richness + total bloom | 25.21 | 3.84 |
| Farm size | 1000m + total bloom | 21.37 | 0 |
|  | 1000m + total bloom + farm size | 23.75 | 2.38 |
| Temperature | **1000m + total bloom** | **21.37** | **0** |
|  | 1000m + total bloom + minimum temperature | 25.57 | 4.20 |
| **Generalist bees** | 1500m | 26.49 | 0 |
| Urbanization predictor | 1000m | 26.49 | 0 |
|  | 2000m | 26.53 | 0.04 |
|  | 500m | 26.63 | 0.14 |
| Floral resource predictor | 1500m + total bloom | 27.00 | 0 |
|  | 1500m + plant spp. richness | 28.81 | 1.80 |
|  | 1500m + plant spp. richness + total bloom | 31.42 | 4.42 |
| Farm size | 1500m + total bloom | 27.00 | 0 |
|  | 1500m + total bloom + farm size | 29.95 | 2.95 |
| Temperature | **1500m + total bloom** | **27.0** | **0** |
|  | 1500m + total bloom + minimum temperature | 28.2 | 1.19 |
| **Specialist bees** | 500m | 31.98 | 0 |
| Urbanization predictor | 1000m | 32.55 | 0.57 |
|  | 1500m | 33.10 | 1.12 |
|  | 2000m | 33.31 | 1.34 |
| Floral resource predictor | 500m + plant spp. | 35.69 | 0 |
|  | 500m + bloom cover | 35.76 | 0.07 |
|  | 500m + plant spp. richness + bloom cover | 40.22 | 4.53 |
| Farm size | 500m + plant spp. richness | 35.69 | 0 |
|  | 500m + plant spp. richness + farm size | 39.11 | 3.42 |
| Temperature | **500m + plant spp. richness** | **35.69** | **0** |
|  | 500m + plant spp. richness + minimum temperature | 40.18 | 4.49 |
| *C. Post-hoc tests on eusocial bees* |  |  |  |
| ***Bombus spp.*** | **1500m** | 31.56 | 0 |
| Urbanization predictor | 2000m | 31.65 | 0.09 |
|  | 1000m | 31.71 | 0.15 |
|  | 500m | 33.42 | 1.86 |
| ***Lasioglossum (Dialictus) spp.*** | **1000m** | 35.95 | 0 |
| Urbanization predictor | 1500m | 36.63 | 0.67 |
|  | 2000m | 36.86 | 0.91 |
|  | 500m | 37.22 | 1.26 |
