# Supplementary material for: The effects of urbanization on bee communities depends on floral resource availability and bee functional traits
Source: PLoS One. 2019 Dec 2;14(12):e0225852. doi: 10.1371/journal.pone.0225852 (PMC6886752; doi:10.1371/journal.pone.0225852)
Supplement: S5 Table — (DOCX) [file pone.0225852.s010.docx]

S5 Table. Pearson correlation tests between minimum site level temperature and impervious surface area.

| Urbanization radius | r | t-value | D.F. | P-value |
| --- | --- | --- | --- | --- |
| 500 m | 0.422 | 1.679 | 13 | 0.117 |
| 1000 m | 0.457 | 1.852 | 13 | 0.087 |
| 1500 m | 0.521 | 2.202 | 13 | 0.046 |
| 2000 m | 0.540 | 2.307 | 13 | 0.068 |
